# Distribution and morphological features of astrocytes and Purkinje cells in the human cerebellum

**DOI:** 10.3389/fnana.2025.1592671

**Published:** 2025-07-04

**Authors:** Christa Hercher, Kristin Ellerbeck, Louise Toutée, Xinyu Ye, Refilwe Mpai, Claudia Belliveau, Maria Antonietta Davoli, W. Todd Farmer, Alanna J. Watt, Keith K. Murai, Gustavo Turecki, Naguib Mechawar

**Affiliations:** ^1^McGill Group for Suicide Studies, Douglas Mental Health University Institute, McGill University, Montreal, QC, Canada; ^2^Integrated Program in Neuroscience, McGill University, Montreal, QC, Canada; ^3^Centre for Research in Neuroscience, Department of Neurology and Neurosurgery, Brain Repair and Integrative Neuroscience Program, The Research Institute of the McGill University Health Centre, Montreal General Hospital, Montreal, QC, Canada; ^4^Department of Biology, McGill University, Montreal, QC, Canada; ^5^Department of Psychiatry, McGill University, Montreal, QC, Canada

**Keywords:** glial fibrillary acidic protein, aldehyde dehydrogenase-1 family member L1, cerebellum, astrocytes, Purkinje cells, stereology, neuroanatomy, microscopy

## Abstract

**Introduction:**

The cerebellar cortex is now recognized as a functionally heterogeneous brain region involved not only in traditional motor functioning but also in higher-level emotional and cognitive processing. Similarly, cerebellar astrocytes also display a high degree of morphological and functional diversity based on their location. Yet, the morphological features and distribution of cerebellar astrocytes have yet to be quantified in the human brain.

**Methods:**

To address this, we performed a comprehensive postmortem examination of cerebellar astrocytes in the healthy human brain using microscopy-based techniques. Purkinje cells (PCs) were also quantified due to their close relationship with Bergmann glia (BG). Using canonical astrocyte markers glial fibrillary acidic protein (GFAP) and aldehyde dehydrogenase-1 family member L1 (ALDH1L1), we first mapped astrocytes within a complete cerebellar hemisphere.

**Results:**

Astrocytes were observed to be differentially distributed across cerebellar layers with their processes displaying known morphological features unique to humans. Stereological quantifications in three functionally distinct lobules demonstrated that the vermis lobule VIIA, folium displayed the lowest densities of ALDH1L1+ astrocytes compared with lobule III and crus I. Assessing cerebellar layers showed that the PC layer had the highest ALDH1L1+ densities while GFAP+ densities and astrocytes colocalizing (ALDH1L1+ GFAP+) were highest in the granule cell layer yet displayed the smallest GFAP-defined territories. PC parameters revealed subtle differences across lobules, with vermis folium VIIA having the lowest PC densities while a trend for the highest BG:PC ratio was observed in the cognitive lobule crus I. Lastly, to determine if these features differ from those of cerebellar astrocytes and PCs in species used to model human illnesses, we performed comparative analyses in mice and macaques showing both divergence and commonalities across species.

**Discussion:**

The present study highlights the heterogeneity of astrocytes in the human cerebellum and serves as a valuable resource on cerebellar astrocyte and PC properties in the healthy human brain.

## 1 Introduction

During evolution, the size and functional heterogeneity of the human cerebellum has expanded in parallel with the neocortex (Barton and Venditti, 2014; Sereno et al., 2020). Imaging studies have largely contributed to the current sophisticated understanding of cerebellar functioning, demonstrating that approximately half of the cerebellar cortex is associated with higher-level cognitive and affective functions (Buckner et al., 2011; Schmahmann et al., 2019; Kawabata et al., 2022). A fundamental three lobe framework indicates that the anterior lobe, containing lobules I–V, are connected to sensorimotor areas of the cerebral cortex and thus associate with traditional cerebellar functions such as motor functioning, gait, posture; the posterior lobe, represented by lobules VI–IX, are involved in cognitive and social behavior tasks associating with prefrontal, parietal association cortices and limbic regions; and the flocculonodular lobe, comprising lobule X and the flocculus and paraflocculus lobules, is phylogenetically the oldest region of the cerebellum responsible for equilibrium, eye movements and adjusting reflexes (Schmahmann et al., 2019). However, recent imaging studies have built upon this framework demonstrating increased complexity of cerebellar functional topography with double and triple representations of sensorimotor, cognitive, and affective functions across multiple lobules and lobes (Xue et al., 2021; Kawabata et al., 2022).

Astrocytes are a highly heterogeneous glial cell population involved in a variety of roles essential for multiple biological functions such as regulating the blood brain barrier through perivascular astrocytic endfeet (Alvarez et al., 2013; Díaz-Castro et al., 2023), modulating synapses and synaptic plasticity through perisynaptic astrocyte processes (Chung et al., 2015; Verkhratsky et al., 2021; Stogsdill et al., 2023), and mediating the transcellular exchange of ions and small metabolites through gap junctions (Mayorquin et al., 2018). Transcriptomics studies have further magnified the diverse signatures of astrocyte subtypes both within and between brain regions (Batiuk et al., 2020; Bayraktar et al., 2020; Endo et al., 2022; Karpf et al., 2022). Within the cerebellum, astrocytes display a high degree of diversity based on the layer they reside in, which likely contributes to their functional range (Cerrato et al., 2018; Matias et al., 2019). For example, velate astrocytes are localized in the granule cell layer (GCL), enwrapping their processes around granule cells and cerebellar glomeruli (Hoogland and Kuhn, 2010). This positioning suggests that velate astrocytes may regulate tissue homeostasis and cerebellar circuit function (Cerrato, 2020). Spanning the Purkinje cell layer (PCL) and molecular layer (ML), Bergmann glia (BG) are specialized astrocytes derived from radial glia with processes in close association with Purkinje cell (PC) dendritic trees and somas. These astrocytes are involved in cerebellar development, PC synaptogenesis, and regulating synaptic activity (Buffo and Rossi, 2013). Scattered non-uniformly throughout the PCL and to varying degrees in the ML, Fañanas cells are little studied and poorly understood cerebellar astrocytes (Goertzen and Veh, 2018). Finally, traditional fibrous astrocytes located in the white matter (WM) align with axons where they are thought to offer structural and metabolic support (Cerrato, 2020). While astrocytes have been elegantly characterized in the cerebral cortex (Colombo and Reisin, 2004; Oberheim et al., 2006; Falcone et al., 2019, 2022; O’Leary et al., 2020; Forrest et al., 2023) few have explored cerebellar astrocytes (Muñoz et al., 2021), with these cells being uncharted in the human cerebellum.

Therefore, this study aimed to understand the morphological diversity and distribution of cerebellar astrocytes and PCs in the healthy human brain. Our results show that glial fibrillary acidic protein (GFAP) immunoreactive (IR) and aldehyde dehydrogenase-1 family member L1 (ALDH1L1)-IR astrocytes are differentially distributed across cerebellar layers with ALDH1L1 being a suitable marker for BG cell bodies and GFAP a robust marker for BG processes and cerebellar fibrous astrocytes in the human cerebellum. Astrocyte processes displayed varicosities and knotted-blebbings with the former being known morphological features unique to the human neocortex. Unbiased stereological quantifications in three functionally distinct lobules demonstrated that the vermis lobule VIIA, folium displayed the lowest densities of ALDH1L1+ astrocytes compared with lobule III and crus I. Examining cerebellar layers showed that ALDH1L1+ densities were highest in the PCL while GFAP+ densities and astrocytes colocalizing (ALDH1L1+ GFAP +) were highest in the GCL. GFAP-defined astrocyte territories were among the largest in the ML and WM while astrocytes in the GCL displayed the smallest territories in the human cerebellum. Quantifying PC parameters revealed that vermis lobule VIIA, folium had the lowest PC densities while a trend for the highest BG:PC ratio was observed in the cognitive lobule crus I. Lastly, to understand if these features differed from those of cerebellar astrocytes and PCs in species used to model human illnesses, we examined these cells in non-human primate macaques and mice showing both structural divergence as well as commonalities across species.

## 2 Materials and methods

### 2.1 Human donors

Postmortem formalin-fixed human cerebella samples from four adults (two males and two females) were obtained from the Douglas-Bell Canada Brain Bank^1^ with no prior history of inflammatory, psychiatric, or neurological disorders prior to death. Following area fraction analysis, vermis lobule VIIA, folium was missing in one subject therefore a replacement subject was used for stereology analysis (Table 1).

**TABLE 1:** Individual demographic information.

Donor	Cause of death	Age (years)	PMI (h)	pH	Analysis
Male 1	Accidental	54	54.5	6.3	SI
Male 2	Accidental	36	49.0	6.3	AF and SI
Male 3	Natural	45	97.5	6.4	AF
Female 1	Natural	54	85.3	6.2	AF and SI
Female 2	Natural	38	43.0	5.9	AF and SI

AF, area fraction; SI, stereology.

### 2.2 Tissue processing

#### 2.2.1 Qualitative and % area coverage assessments

**FIGURE 1:**
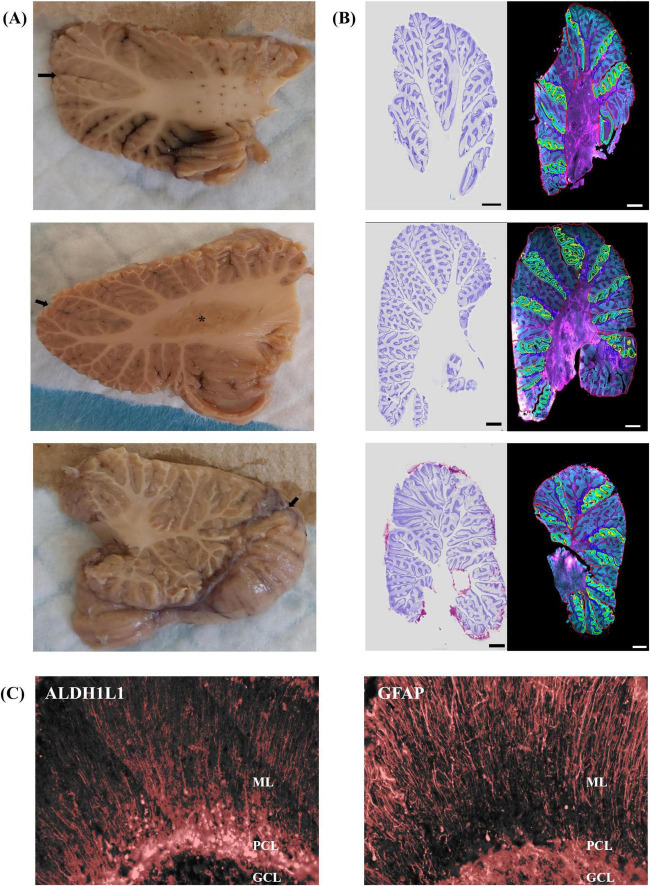
Representative sagittal slabs that were dissected and immunolabeled from the human cerebellum. **(A,B)** Top panel represents lateral position, *X* = 26, middle panel illustrates the DCN at the level of the dentate nucleus *X* = 20, bottom panel displays the vermis *X* = 6. **(A)** The lateral surface is shown in top and middle panels, and the medial surface is shown in the bottom panel to display the entire vermis. Black arrows point to great horizontal fissure. Asterisk labels dentate nucleus. **(B)** Left image displays Nissl stained section for histological reference. Right image displays immunolabeling for GFAP astrocytes in magenta and ALDH1L1 astrocytes in cyan. Lobules and layers were annotated and outlined using QuPath *with red outlining lobules, cyan outlines ML, green denotes PCL, yellow GCL, and blue outlines WM.* MRI Atlas of the human cerebellum. Schmahmann et al. (2000) acted to guide lateral (*X* = 26), DCN (*X* = 20), and vermis (*X* = 6). Scale bar 5 mm. **(C)** Threshold representations of % area coverages in the human cerebellum for ALDH1L1 (left image) and GFAP (right image) immunoreactive astrocytes. Red overlay indicates pixels included in the analysis which were based on identifying the mean intensity and standard deviation of our signals of interest. Right hemisphere is shown. ALDH1L1, aldehyde dehydrogenase-1 family member L1; DCN, deep cerebellar nuclei; GCL, granule cell layer; GFAP, glial fibrillary acidic protein; ML, molecular layer; PCL, Purkinje cell layer; WM, white matter.

Notably, 2” × 3” human sagittal cerebellar slabs comprising the complete right hemisphere were dissected at three anatomical levels: lateral, deep cerebellar nuclei (DCN), and vermis (Figure 1A), following the atlas of Schmahmann et al. (2000). Slabs were suspended in a 30% sucrose solution until equilibrium was reached followed by flash freezing in −35°C isopentane. At each anatomical level, cerebellar hemispheres were sectioned at 30 μm in the sagittal plane, mounted on 2” × 3” glass slides, dried overnight at room temperature (RT) followed by immediate immunolabeling.

#### 2.2.2 Quantitative stereology

Notably, 1 cm^3^ tissue dissections from formalin fixed sagittal slabs were taken at the level of the dentate nuclei (lobule III and crus I, *X* = 20, Schmahmann et al., 2000) and in the vermis (folium VIIA, *X* = 6, Schmahmann et al., 2000) and prepared as above. These lobules were chosen as each represents a unique functional domain: the vermis associating with emotional regulation, crus I with cognition, and lobule III with traditional cerebellar motor functions (Buckner et al., 2011; Schmahmann, 2019). Notably, 1 cm^3^ human tissue blocks were then systematically and exhaustively sectioned into 30 μm-thick sagittal sections, mounted on Superfrost glass slides, and stored at −80°C until immunostaining. An equally spaced series of sections per lobule of interest were immunolabeled (4–7 sections/subject/anatomical position). Section sampling followed systematic and random sampling principles of stereology (Mouton, 2002; Kreutz and Barger, 2018).

### 2.3 Immunolabeling

Sections were air-dried and heated in a 60°C oven for 30 min to aid in section adherence to the glass slides. Sections were then cooled for 5 min at RT followed by rinsing in phosphate-buffered saline (PBS) for 5 min. Antigen retrieval was performed using proteinase K (1:1,000) for 15 min followed by PBS washes. Sections were blocked in 10% normal donkey serum (NDS, Jackson ImmunoResearch Labs, Cat# 017-000-121, RRID:AB_2337258) and PBS for 1 h at RT. Human cerebellar sections were incubated overnight at 4°C in chicken polyclonal anti-GFAP (1:1,000, Abcam, Cat# ab4674, RRID:AB_304558) and mouse monoclonal anti-ALDH1L1 (1:250, Millipore, Cat# MABN495, RRID:AB_2687399) in blocking solution. Following primary antibody incubations, sections were rinsed in PBS followed by application of secondary antibodies, Alexa Fluor^®^ 488-conjugated donkey anti-chicken (1:500, Jackson ImmunoResearch Labs, Cat# 703-545-155, RRID:AB_2340375) and Alexa Fluor^®^ 647-conjugated donkey anti-mouse (1:500, Jackson ImmunoResearch Labs, Cat# 715-605-151, RRID:AB_2340863) for 1 h at RT. Following PBS washes, sections were quenched for autofluorescence using TrueBlack^®^ (Biotium, 23007) for 75 s, rinsed and coverslipped with Vectashield Vibrance with DAPI mounting medium (Vector, H-1800).

### 2.4 Image analysis

#### 2.4.1 Qualitative and % area coverage assessments

Three sections containing the complete right cerebellar hemisphere were sampled representative of each anatomical position: lateral, DCN level, and vermis level. An additional three sections, adjacent to the immunofluorescent sections, were stained for cresyl violet (Nissl stain) and acted as a histological guide. Slides were scanned on an Evident Scientific VS120 slide scanner at 20×. Exposure times remained constant for all sections. Cerebellar layers, ML, PCL, GCL, and WM, in all cerebellar lobules were outlined and annotated using QuPath (Bankhead et al., 2017, version 0.3.2, RRID:SCR_018257) guided closely by a reference atlas (Schmahmann et al., 2000). One complete folia per lobule was chosen for annotations in order to maximize efficiency (Figure 1B). Percent area coverage for GFAP-IR and ALDH1L1-IR astrocytes in each layer of each lobule were obtained using a threshold approach based on the mean intensity and standard deviation of our signal of interest. Pixels at and above the threshold value were considered positive while pixels below the threshold were reflected as background (Figure 1C).

#### 2.4.2 Quantitative stereology assessments

Unbiased stereology was performed using the software Stereo Investigator (MicroBrightfield, Inc., RRID:SCR_004314, United States, Stereo Investigator, RRID:SCR_002526). Image stacks were acquired every 2 μm throughout our mounted tissue thickness using a Zeiss ApoTome2 Axio Imager.M2 microscope system at 63× (N.A 1.4) in each lobule and layer of interest. Tissue thickness was measured at every sampling site to account for wavy tissue and to ensure optimal image quality. The optical fractionator Probe was applied to the image stacks using a 9 μm dissector height with 1 μm guard zones. Unbiased estimates of ALDH1L1+ and GFAP+ astrocytes were quantified in the PCL, GCL, and WM. In the PCL, the nucleator probe (isotropic sampling, four rays) was applied to the image stacks where for each donor, 60 PC body and nucleus sizes were measured in each lobule of interest. PCs were counted directly using the optical fractionator probe in the PCL to minimize imaging times. Unbiased volume estimates were generated by applying the Cavalieri probe on the same contours used for counting in each layer. Coefficients of error (Gunderson *m* = 1) ≤ 0.1 were achieved to ensure accurate sampling of our stereological estimates (Supplementary Table 1).

### 2.5 Animal samples

**FIGURE 2:**
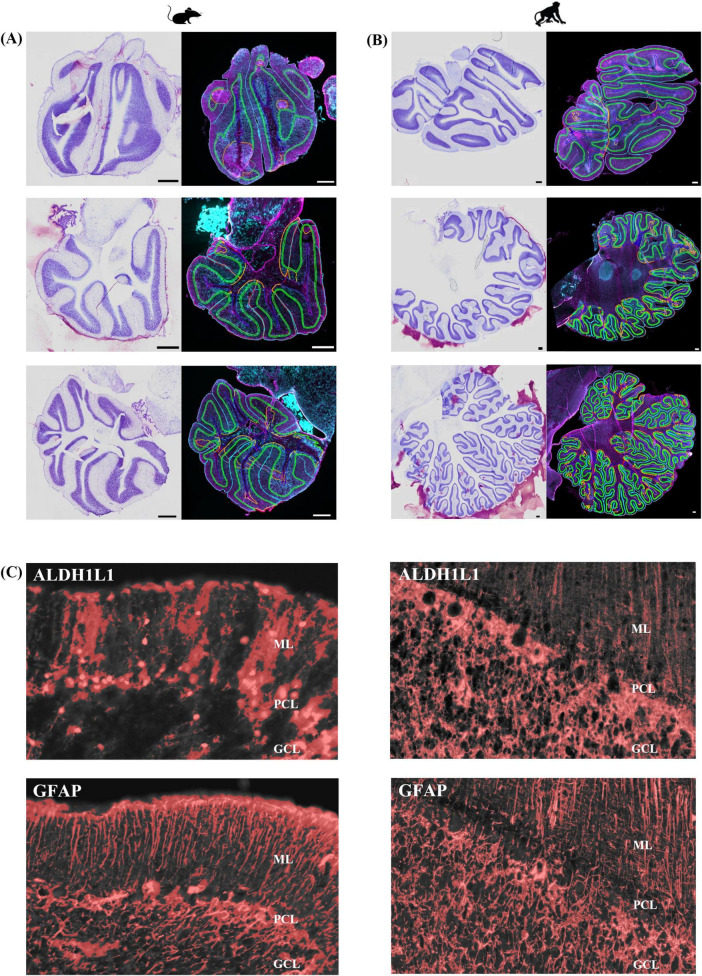
Representative sagittal images at three anatomical positions in mouse and macaque cerebella. **(A,B)** Top panel represents lateral position, middle panel illustrates the DCN at the level of the dentate nucleus, bottom panel displays the vermis. Nissl-stained sections (left image) acted as a histological reference. Right image displays immunolabeling for GFAP-IR astrocytes in magenta and ALDH1L1-IR astrocytes in cyan. Lobules and layers were annotated and outlined using QuPath. Allen P56 Mouse Brain Atlas, sagittal images were used to guide lateral (reference image 3), DCN (reference image 7), and vermis (reference image 17) mice sections. Cerebellum of the rhesus monkey: atlas of lobules, laminae, and folia, in sections, Madigan and Carpenter (1971) was used to guide lateral (figures 37–38), DCN (figures 27–28), and vermis (figures 15–16) macaque sections. Scale bar 500 μm. **(C)** Threshold representations of % area coverages for ALDH1L1-IR (top images) and GFAP-IR (bottom images) astrocytes in mouse and macaque cerebella. Red overlay indicates pixels included in the analysis which were based on identifying the mean intensity and standard deviation of our signals of interest. Note: tdTomato reporter signal was used to visualize mouse ALDH1L1-IR astrocytes. ALDH1L1, aldehyde dehydrogenase-1 family member L1; DCN, deep cerebellar nuclei; GCL, granule cell layer; GFAP, glial fibrillary acidic protein; IR, immunoreactive; ML, molecular layer; PCL, Purkinje cell layer.

Cerebella were acquired from two adult male cynomolgus macaques (generous gift from Dr. Michael Petrides) and from two adult male Aldh1L1-Cre/ERT2; Rosa26-TdTomato transgenic mice induced with tamoxifen. Aldh1L1-Cre/ERT2; Rosa26-TdTomato transgenic mice induced with tamoxifen were needed because ALDH1L1 immunolabeling is particularly challenging in mouse brain tissue (unpublished observations; O’Leary et al., 2020). Transgenic mice were generated by crossing Aldh1L1-Cre/ERT2 mice (JAX stock no. 031008) with Ai9 Rosa26-TdTomato reporter line mice (JAX stock no. 007909) (Madisen et al., 2010; Srinivasan et al., 2016). This specific transgenic Cre reporter line, has previously been reported to show about an 85% overlap with astrocytes immunolabeled with S100β in the cerebellum (Winchenbach et al., 2016). The tamoxifen solution for *in vivo* activation of Cre recombinase was prepared by dissolving tamoxifen (Sigma) in 100% EtOH at 200 mg/ml by gentle heating. Once the tamoxifen was in solution, it was diluted in corn oil (1:10; Sigma). The resulting 20 mg/ml solution was delivered through intraperitoneal (IP) injections of adult mice (5 weeks+; 1 injection per day for 5 days). Breeding and animal procedures were carried out with the approval of the McGill Animal Care Committee in accordance with the Canadian Council on Animal Care guidelines. Mice and macaques were perfused intracardially with ice-cold PBS followed by 4% formaldehyde in 0.1 M phosphate buffer. Brains were rapidly removed and postfixed in 10% neutral buffered formalin followed by separation of the cerebella from the cerebrums. Cerebella were suspended in a 30% sucrose solution until equilibrium was reached followed by rapid freezing in −35°C isopentane. Cerebellar hemispheres were systematically sectioned into 30 μm-thick sagittal sections, mounted on Superfrost glass slides, and stored at −80°C until immunostaining. For quantitative stereology, three series of sections were sampled based on anatomical position, one containing lobule III, one encompassing crus I, and a final series was taken throughout the vermis, guided by corresponding atlases (Allen Institute, 2004; Madigan and Carpenter, 1971). A total of 3–7 sections/animal/region of interest were immunolabeled. Immunolabeling protocols were optimized in each species to ensure that the antibodies stained the appropriate pattern of cellular morphology as previously demonstrated (O’Leary et al., 2020). Sections were air-dried and heated in a 60°C oven for 15 min, cooled for 5 min at RT followed by rinsing in PBS for 5 min. Antigen retrieval was performed using proteinase K (1:1,000) for 15 min followed by PBS washes. Sections were blocked in 10% NDS (Jackson ImmunoResearch Labs, Cat# 017-000-121, RRID:AB2337258) and PBS+ 0.2% Triton-X for 1 h at RT. Mouse sections were incubated overnight at 4°C in chicken polyclonal anti-GFAP (1:250, Abcam, Cat# ab4674, RRID:AB_304558) and blocking solution. Macaque sections were incubated for 48 h at 4°C in chicken polyclonal anti-GFAP (1:250, Abcam, Cat# ab4674, RRID:AB_304558) and mouse monoclonal anti-ALDH1L1 (1:50, Millipore, Cat# MABN495, RRID:AB_2687399) in blocking solution. Following primary antibody incubations, sections were rinsed in PBS followed by application of secondary antibodies, Alexa Fluor^®^ 488-conjugated donkey anti-chicken (1:500, Jackson ImmunoResearch Labs, Cat# 703-545-155, RRID:AB_2340375, mouse and macaque sections) and Alexa Fluor^®^ 647-conjugated donkey anti-mouse (1:500, Jackson ImmunoResearch Labs, Cat# 715-605-151, RRID:AB_2340863, macaque sections) for 1 h at RT. Following PBS washes, sections were quenched for autofluorescence using TrueBlack^®^ (Biotium, 23007) for 75 s, rinsed and coverslipped with Vectashield Vibrance with DAPI mounting medium (Vector, H-1800). Qualitative, % area coverage, and stereology assessments were performed as described above in human samples with reference atlases for mice (Allen P56 Mouse Brain Atlas, sagittal) and macaques (Madigan and Carpenter, 1971) acting as guides (Figure 2; see Supplementary Table 2 for stereological parameters).

To better understand the presence and absence of astrocytes observed in the mouse DCN, sections containing the fastigial nucleus were subsequently immunolabeled for qualitative purposes with primary antibodies rabbit monoclonal anti-sox9 (1:1,000, Abcam, Cat# ab185966, RRID:AB_2728660), mouse monoclonal anti-100β (1:1,000, Sigma-Aldrich, Cat# S2532, RRID:AB_477499), and mouse monoclonal anti-glutamine synthetase (1:500, Millipore, Cat# MAB302, RRID:AB_2110656) following the above protocol.

### 2.6 Statistical analysis

Statistical analyses and graphical representations were performed using SAS JMP Pro 18 (SAS Institute, Cary, NC, USA, RRID:SCR_022199) and applied to the human cerebellar datasets only. Distributions were assessed with Shapiro–Wilk tests and by examining normal quantile plots. Spearman correlations assessed the relationship between dependent variables and covariates [age, postmortem interval (PMI), and pH] and were included as covariates for the significant relationships. ALDH1L1-IR and GFAP-IR % area coverages were analyzed using mixed-effects models with layer and either anatomical position, lobule or lobe as fixed factors, followed by Tukey’s honestly significant difference test. To test if there were differences between astrocyte markers by cerebellar layers, a mixed-effects model was used with astrocyte marker and cerebellar layers as fixed factors followed by Tukey’s honestly significant difference test. The DCN was treated as a separate region where one-way analysis of variance (ANOVA) models were constructed for ALDH1L1-IR and GFAP-IR % area coverages with anatomical position as a fixed factor. A one-way ANOVA was used to compare % area coverage by astrocyte marker in the DCN. Astrocyte densities were analyzed using mixed-effects models with layer and lobule as fixed factors, followed by Tukey’s honestly significant difference test. PC parameters were analyzed using one-way ANOVAs, followed by Tukey’s honestly significant difference test. The significance threshold was set at 0.05 and all data presented are mean ± SEM.

## 3 Results

### 3.1 Differential immunoreactivity of canonical astrocyte markers GFAP and ALDH1L1 across cerebellar layers

**FIGURE 3:**
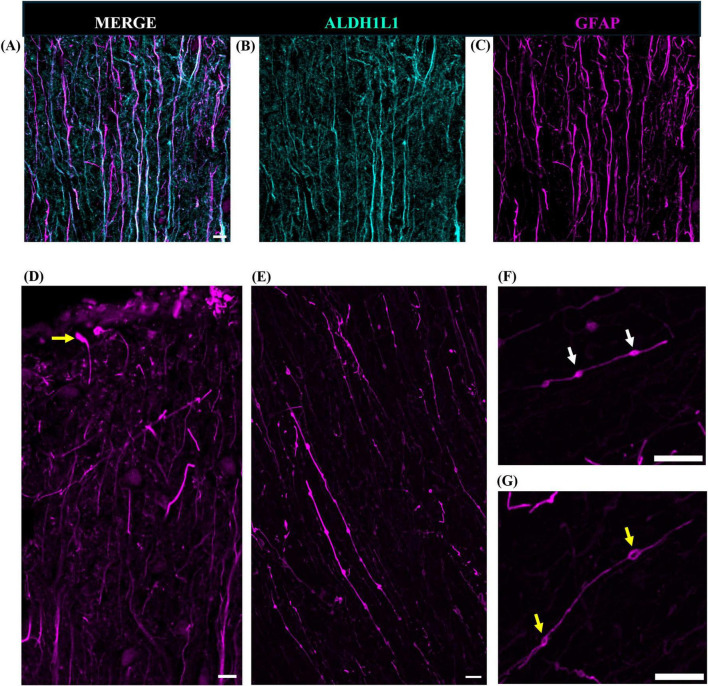
Human cerebellar astrocytes in the molecular layer. ALDH1L1-IR and GFAP-IR processes (**B,C**, respectively) displayed complex horizontal branching with lateral appendages highly visible. Bulbous astrocytic end feet (yellow arrow) appeared sparse in humans **(D)**. Along GFAP-IR processes, varicosity like protrusions (**E,F**, white arrows) and knotted-blebbings (**E,G**, yellow arrows) were observed. Panels **(A–D)** were acquired using a Zeiss ApoTome2 Axio Imager.M2 microscope system, 40×. Panels **(E–G)** were acquired on an Evident Scientific FV1200 confocal microscope system, 60×. Scale bar 10 μm. ALDH1L1, aldehyde dehydrogenase-1 family member L1; GFAP, glial fibrillary acidic protein; IR, immunoreactive.

**FIGURE 4:**
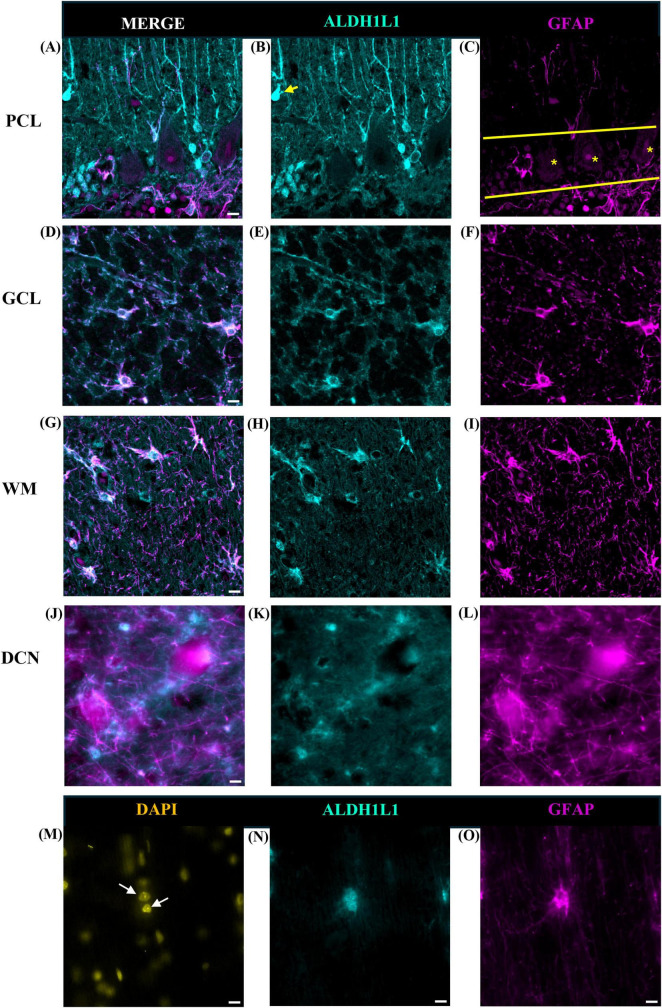
Astrocytes in the human cerebellum. ALDH1L1 strongly labeled Bergmann glia in the PCL **(A–C)**. Labeling of Fañanas cells, or possibly displaced Bergmann glia were also observed (**B**, yellow arrow). Yellow solid line outlines Purkinje cell layer and yellow asterisks labels Purkinje cells **(C)**. In the GCL **(D–F)** astrocyte cell bodies showed robust ALDH1L1-IR labeling **(E)** while GFAP was highly visible in astrocytic processes **(F)**. Typical fibrous astrocytes were highly visible in the WM **(G–I)**. In the DCN astrocyte cell bodies were difficult to distinguish **(J–L)**. Fastigial nucleus is shown. WM astrocytes in close proximity (possibly twin cells) were also observed **(M–O)**. White arrows point to nuclei of possible twin cells **(M)**. Images were acquired using a Zeiss ApoTome2 Axio Imager.M2 microscope system, 40×. Scale bar 10 μm. ALDH1L1, aldehyde dehydrogenase-1 family member L1; DCN, deep cerebellar nuclei; GCL, granule cell layer; GFAP, glial fibrillary acidic protein; IR, immunoreactive; PCL, Purkinje cell layer; WM, white matter.

We used two canonical astrocyte markers, ALDH1L1 and GFAP, since each marker labels different subsets of astrocytes, to visualize astrocytes in the cerebellar cortex and DCN. We found that the ML of the cerebellum was largely comprised of GFAP-IR processes with few astrocytic cell bodies (Figures 3A–C). While we did observe several ALDHL1+ GFAP-cells in the lower half of the ML, it was difficult to state whether these were Fañanas cells or simply displaced BG cells (Figure 4B). Further labeling with Kv2.2 potassium channel and calsenilin could help disentangle these astrocyte classes (Goertzen and Veh, 2018). ML processes were complex, displaying a high degree of branched and horizontal processes (Figures 3A–C). In the upper limit of the ML, GFAP-IR bulbous endfeet were difficult to visualize appearing sparse (Figure 3D). Varicosity-like protrusions, an astrocytic feature unique to the neocortex of human and non-human primates, were also observed along ML processes in some of the human cerebellar samples (Figures 3E, F). Additionally, knotted blebbing’s along these ML processes were also visible (Figure 3G). The PCL contained mainly ALDH1L1-IR cell bodies with these astrocytes being in close proximity to PCs (Figures 4A–C). Despite the density of granule cells, velate astrocytes were highly visible in the GCL. A dense cell body labeling was observed with ALDH1L1 and GFAP, with GFAP extending further along astrocyte processes (Figures 4D–F). Characteristic fibrous astrocytes were observed in the WM with GFAP-IR being particularly intense in these cells (Figures 4G–I). Aligning with a previous report in the cerebral cortex (O’Leary et al., 2020), we also noted astrocytes in close proximity, possibly indicative of twin cell, in human cerebellar WM (Figures 4M–O). We also examined astrocytes within the DCN and found it difficult to distinguish cell bodies from processes (Figures 4J–L).

Overall, these qualitative observations indicate layer-specific preferences for astrocytic markers particularly highlighting ALDH1L1 as a suitable and robust marker for BG in the human cerebellum.

### 3.2 Divergence of ALDH1L1 and GFAP astrocytic % area coverages within the human cerebellum

**FIGURE 5:**
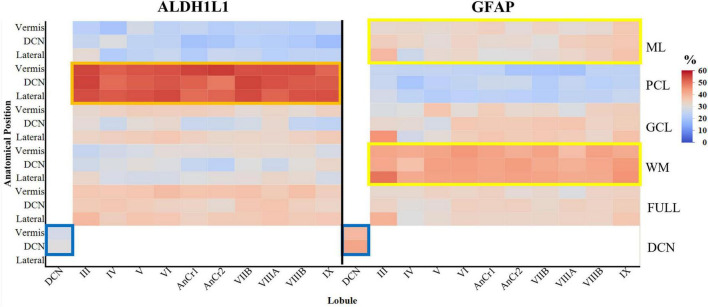
Area coverages of ALDH1L1-IR and GFAP-IR astrocytes in cerebellar layers and lobules in the human cerebellum (percentages are reported). ALDH1L1 coverages were significantly higher in the PCL compared to GFAP, suggesting a high expression of this astrocytic marker in Bergmann glia (orange rectangle). GFAP coverages were significantly higher than ALDH1L1 coverages in both the ML and WM, indicating that GFAP is a robust marker for Bergmann glia processes and cerebellar fibrous astrocytes respectively (yellow rectangles). Within the DCN, GFAP coverages were significantly higher than ALDH1L1 coverages (blue rectangles). Full indicates average of all cerebellar layers excluding the DCN. ALDH1L1, aldehyde dehydrogenase-1 family member L1; DCN, deep cerebellar nuclei; GCL, granule cell layer; GFAP, glial fibrillary acidic protein; IR, immunoreactive; ML, molecular layer; PCL, Purkinje cell layer; WM, white matter.

To gain a foundational quantitative understanding of the distribution of cerebellar astrocytes, we next assessed whether the area fraction of astrocytes differed within the human cerebellum. To address this, we obtained % area coverages for ALDH1L1-IR and GFAP-IR cerebellar astrocytes in each layer, of every lobule, at three different anatomical position (lateral, at the level of the DCN, and in the vermis) in a complete hemisphere of the human cerebellum (Figure 5). Age was positively corrected with GFAP area coverages and was included as a covariate in GFAP analyses (Spearman ρ = 0.1214, *p* = 0.0095).

#### 3.2.1 Overall astrocyte % area coverages

The % area coverages of all labeled astrocytes (ALDH1L1-IR and GFAP-IR combined) in a complete cerebellar hemisphere were highest in the PCL (39%) followed by WM (36%), GCL (33%), and lowest in the ML (28%), [main effect of layer *F*(3,17.4) = 104.5229, *p* < 0.0001]. We did not observe a main effect of anatomical position (lateral, DCN, and vermis) [*F*(2,6.0) = 0.7084, *p* = 0.5294] or a layer × anatomical position interaction [*F*(6,17.5) = 0.8847, *p* = 0.5266]. In the DCN, % area coverages of astrocytes did not differ between the dentate nucleus (37%) and vermis fastigial nucleus (33%) [*F*(2,9.0) = 0.1823, *p* = 0.8363]. We next evaluated astrocytic area coverages in each cerebellar lobule and layer observing small discrepancies across the lobules [lobule × layer interaction, *F*(27,46.8) = 0.6330, *p* = 0.8974; main effect of lobule *F*(9,20.5) = 0.7685, *p* = 0.6462]. With the knowledge that there are lobule specific functions within the cerebellum where a simplistic model classifies anterior lobules I–V as exhibiting traditional motor functions, posterior lobules (VI–IX) associating with cognitive and emotional regulation, and the flocculonodular lobe (X) responsible for equilibrium, eye movements and adjusting reflexes, we explored whether the current astrocyte markers differed according to these functional domains in lateral, DCN, and vermis regions (Buckner, 2013; Klein et al., 2016). We did not observe a functional lobe × layer interaction [*F*(6,207.6) = 0.2752, *p* = 0.9481] or an effect of functional lobe [*F*(2,20.3) = 0.1468, *p* = 0.8644] on % area coverages of cerebellar astrocytes.

#### 3.2.2 ALDH1L1-IR % area coverages

The % area coverages of ALDH1L1-IR astrocytes in a complete cerebellar hemisphere were highest in the PCL (53%) followed by GCL (32%), WM (30%), and lowest in the ML (23%), [main effect of layer *F*(3,20.6) = 383.3021, *p* < 0.0001]. We did not observe a main effect of anatomical position (lateral, DCN, and vermis) [*F*(2,7.2) = 2.7412, *p* = 0.1300] or a layer × anatomical position interaction [*F*(6,20.6) = 1.5744, *p* = 0.2047]. In the DCN, % area coverages of ALDH1L1-IR astrocytes did not differ between the dentate nucleus (30%) and vermis fastigial nucleus (28%) [*F*(1,4.0) = 0.8243, *p* = 0.4153]. ALDH1L1-IR astrocytic area coverages did not differ across cerebellar lobules and layers [lobule × layer interaction, *F*(27,73.7) = 0.8289, *p* = 0.7014; main effect of lobule *F*(9,27.3) = 0.5582, *p* = 0.8187]. We did not observe a functional lobe × layer interaction [*F*(6,27.9) = 0.7454, *p* = 0.6180] or an effect of functional lobe [*F*(2,10.4) = 0.9220, *p* = 0.4279] on ALDH1L1-IR % area coverages of cerebellar astrocytes.

#### 3.2.3 GFAP-IR % area coverages

Glial fibrillary acidic protein immunoreactive astrocyte coverages diverged from those of ALDH1L1-IR coverages across cerebellar layers within a complete hemisphere. GFAP-IR % area coverages were highest in the WM (43%) followed by GCL (34%) and ML (33%), and lowest in the PCL (24%) [main effect of layer *F*(3,20.5) = 165.6406, *p* < 0.0001]. We did not observe a main effect of anatomical position (lateral, DCN, and vermis) [*F*(2,5.3) = 0.1224, *p* = 0.8872] or a layer × anatomical position interaction [*F*(6,20.5) = 0.2807, *p* = 0.9395]. In the DCN, GFAP-IR astrocyte coverages did not differ between the dentate nucleus (43%) and vermis fastigial nucleus (40%) [*F*(2,3.0) = 0.4599, *p* = 0.6696]. GFAP-IR astrocytic area coverages did not differ across cerebellar lobules and layers [lobule × layer interaction, *F*(27,56.3) = 1.1136, *p* = 0.3580; main effect of lobule *F*(9,22.3) = 1.6139, *p* = 0.1716]. We did not observe a functional lobe × layer interaction [*F*(6,38.4) = 1.5575, *p* = 0.1860] or an effect of functional lobe [*F*(2,7.7) = 2.3999, *p* = 0.1546] on GFAP-IR % area coverages of cerebellar astrocytes.

Taken together, we observed differences in coverages of our canonical astrocyte markers across cerebellar layers, cerebellar layer × astrocyte marker interaction [*F*(3,16.6) = 342.9722, *p* < 0.0001] with ALDH1L1 coverages being significantly higher in the PCL compared to GFAP (Tukey’s multiple comparison test, *p* < 0.0001), suggesting a high expression of this astrocytic marker in BG. However, in both the ML and WM, GFAP coverages were significantly higher than ALDH1L1 coverages (Tukey’s multiple comparison test, *p* < 0.0001), indicating that GFAP is a robust marker for BG processes and cerebellar fibrous astrocytes respectively. In the GCL, ALDH1L1 and GFAP coverages were equally represented. Within the DCN, GFAP coverages were significantly higher than ALDH1L1 coverages [41% vs. 29%, respectively, *F*(2,9.0) = 10.1566, *p* = 0.0049].

### 3.3 Layer specific heterogeneity of cerebellar astrocyte densities

Building on the above knowledge that ALDH1L1 and GFAP % area coverages in the human cerebellum display layer specific heterogeneity, we next aimed to quantify these cells utilizing robust quantitative stereological principles in three functionally distinct lobules of interest: lobule III motor, crus I cognitive, and vermis lobule VIIA, folium emotion. Based on our qualitative and semi-quantitative observations, we chose to focus our quantifications on cerebellar layers as it was difficult to visualize astrocyte cell bodies in DCN.

#### 3.3.1 Overall astrocyte densities

Astrocyte densities (ALDH1L1+ and GFAP+ combined) in the PCL were lowest in the vermis compared to lobule III (29%, *p* = 0.0259) and crus I (33%, *p* = 0.0025) [layer × lobule interaction *F*(4,16.0) = 4.9327, *p* = 0.0088 followed by Tukey’s multiple comparison test, main effect of lobule: *F*(2,8.0) = 6.2151, *p* = 0.0235, 35% decrease in vermis vs. lobule III, *p* = 0.0358; 32% decrease vs. crus I, *p* = 0.0443; main effect of layer: *F*(2,16.0) = 308.3662, *p* < 0.0001, 79% increase PCL astrocyte densities vs. GCL densities, *p* < 0.0001; 94% increase PCL astrocyte densities vs. WM densities, *p* < 0.0001; 63% increase GCL astrocyte densities vs. WM astrocyte densities, *p* = 0.0088].

#### 3.3.2 ALDH1L1+ astrocyte densities

**FIGURE 6:**
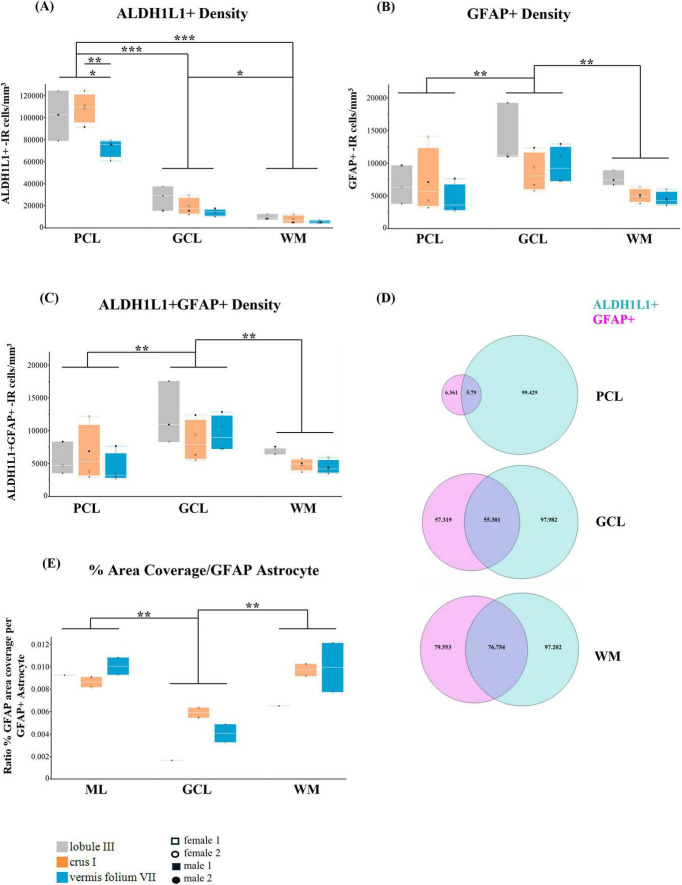
Heterogeneity of cerebellar astrocytes in the human cerebellum. **(A)** ALDH1L1+-IR (immunoreactive) cells/mm^3^. In the PCL, ALDH1L1+ densities were the lowest in the vermis compared to lobule III (29%, *p* = 0.0254) and crus I (33%, *p* = 0.0021). Irrespective of layer, the vermis had the lowest densities (34% decrease in vermis vs. lobule III, *p* = 0.0354; 32% decrease vs. to crus I, *p* = 0.0359). Irrespective of lobule, the PCL had the highest ALDH1L1+ densities while the WM had the lowest (79% increase PCL ALDH1L1+ densities vs. GCL ALDH1L1+ densities, *p* < 0.0001; 92% increase PCL ALDH1L1+ densities vs. WM ALDH1L1+ densities, *p* < 0.0001; 63% increase GCL ALDH1L1+ densities vs. WM ALDH1L1+ densities, *p* = 0.0101. **(B)** GFAP+-IR cells/mm^3^. GFAP+ densities were highest in the GCL compared to PCL (42% increase, *p* = 0.0028) and WM (46% increase GCL GFAP+ densities vs. WM GFAP+ densities, *p* = 0.0017). **(C)** ALDH1L1+ GFAP+-IR cells/mm^3^. ALDH1L1+ GFAP+ astrocyte densities were highest in the GCL (45% increase vs. PCL ALDH1L1+ GFAP+ densities, *p* = 0.0011; 46% increase GCL ALDH1L1+ GFAP+ densities vs. WM ALDH1L1+ GFAP+, *p* = 0.0012). **(D)** Percentage of ALDH1L1+ and GFAP+ astrocytes across cerebellar layers showed that most astrocytes in the human cerebellum were ALDH1L1+. **(E)** GFAP-defined territories (percentage per GFAP+ astrocyte). GFAP+ astrocyte territories were smallest in the GCL (73% smaller than Bergmann glia in the ML, *p* = 0.0026; 72% smaller than WM fibrous astrocytes, *p* = 0.0040). ALDH1L1, aldehyde dehydrogenase-1 family member L1; GCL, granule cell layer; GFAP, glial fibrillary acidic protein; PCL, Purkinje cell layer; WM, white matter. *p < 0.05, ^**^p < 0.01, ^***^p < 0.001.

Similarly for ALDH1L1+ astrocyte densities, the PCL displayed the lowest densities in the vermis compared to lobule III (29%, *p* = 0.0254) and crus I (33%, *p* = 0.0021) [layer × lobule interaction *F*(4,16.0) = 4.8275, *p* = 0.0096; main effect of lobule: *F*(2,8.0) = 6.5205, *p* = 0.0209, 34% decrease in vermis vs. lobule III, *p* = 0.0354; 32% decrease vs. crus I, *p* = 0.0359; main effect of layer: *F*(2,16.0) = 310.4091, *p* < 0.0001, 79% increase PCL ALDH1L1+ densities vs. GCL ALDH1L1+ densities, *p* < 0.0001; 92% increase PCL ALDH1L1+ densities vs. WM ALDH1L1+ densities, *p* < 0.0001; 63% increase GCL ALDH1L1+ densities vs. WM ALDH1L1+ densities, *p* = 0.0101] (Figure 6A).

#### 3.3.3 GFAP+ astrocyte densities

Within the human cerebellum, the distribution of GFAP+ astrocyte densities differed from that of ALDH1L1+ astrocyte densities. GFAP+ densities were highest in the GCL [main effect of layer: *F*(2,16.0) = 11.3699, *p* = 0.0008], 42% increase GCL GFAP+ densities vs. PCL GFAP+ densities, *p* = 0.0028; 46% increase GCL GFAP+ densities vs. WM GFAP+ densities, *p* = 0.0017 (Figure 6B). We did not observe a significant main effect of lobule *F*(2,8.0) = 2.3933, *p* = 0.1532 or a layer × lobule interaction *F*(4,16.0) = 1.0898, *p* = 0.3947.

#### 3.3.4 ALDH1L1+ GFAP+ astrocyte densities

ALDH1L1+ GFAP+ astrocyte densities were highest in the GCL [main effect of layer: *F*(2,16.0) = 13.1252, *p* = 0.0004], 45% increase GCL ALDH1L1+ GFAP+ densities vs. PCL ALDH1L1+ GFAP+ densities, *p* = 0.0011; 46% increase GCL ALDH1L1+ GFAP+ densities vs. WM ALDH1L1+ GFAP+, *p* = 0.0012] (Figure 6C). We did not observe a significant main effect of lobule *F*(2,8.0) = 1.1834, *p* = 0.3546 or a layer × lobule interaction *F*(4,16.0) = 0.9524, *p* = 0.4597.

Overall, in the human cerebellum, the vermis lobule VIIA, folium displayed the lowest ALDH1L1+ densities compared with lobule III and crus I. We observed the highest ALDH1L1+ densities in the PCL while GFAP+ densities and astrocytes colocalizing (ALDH1L1+ GFAP+) were highest in the GCL. Consistent with the notion that ALDH1L1 acts as a pan astrocytic marker, we found that most astrocytes in the human cerebellum were ALDH1L1+ (Figure 6D).

### 3.4 GFAP-defined territories differ across layers in the human cerebellar cortex

A hallmark of astrocytes is their complex morphology comprised of intricate fine processes which interact with a host of crucial biological functions (Mayorquin et al., 2018; Díaz-Castro et al., 2023; Stogsdill et al., 2023). Recent reports have suggested that regions displaying smaller astrocyte territories compensate with higher astrocyte densities (Viana et al., 2023). Therefore, it was of interest to explore whether density and size were related in our datasets, particularly for GFAP-IR astrocytes. Combining our measurements of % area coverage together with stereological estimates for astrocyte densities, we calculated the % area coverage per GFAP+ astrocyte yielding 2D estimates of GFAP-defined territories. GFAP was chosen as the astrocytic marker of choice as it provides extensive labeling of the astrocyte processes compared to ALDH1L1. We included only those subjects where both the lobule specific % area coverage data and density counts were available. We observed GFAP+ astrocyte territories to be significantly smaller in the GCL [main effect of layer *F*(2,4.0) = 41.4055, *p* = 0.0021]; followed by Tukey’s multiple comparison test showing 73% smaller GFAP+ territories in the GCL vs. BG (ML) GFAP+ territories (*p* = 0.0026), 72% smaller GFAP+ territories in the GCL vs. fibrous astrocyte (WM) GFAP+ territories (*p* = 0.0040). There was not an effect of lobule *F*(2,2.0) = 1.2728, *p* = 0.4400 or a layer × lobule interaction *F*(4,4.0) = 2.8342, *p* = 0.1686 (Figure 6E).

Overall, in the human cerebellum, while we observed the highest GFAP+ astrocyte densities in the GCL, these astrocytes displayed the smallest territories. Similarly, GFAP+ astrocyte territories were the largest in the ML and WM, with the latter displaying the lowest GFAP+ astrocyte densities.

### 3.5 Purkinje cell parameters in the human cerebellum

**FIGURE 7:**
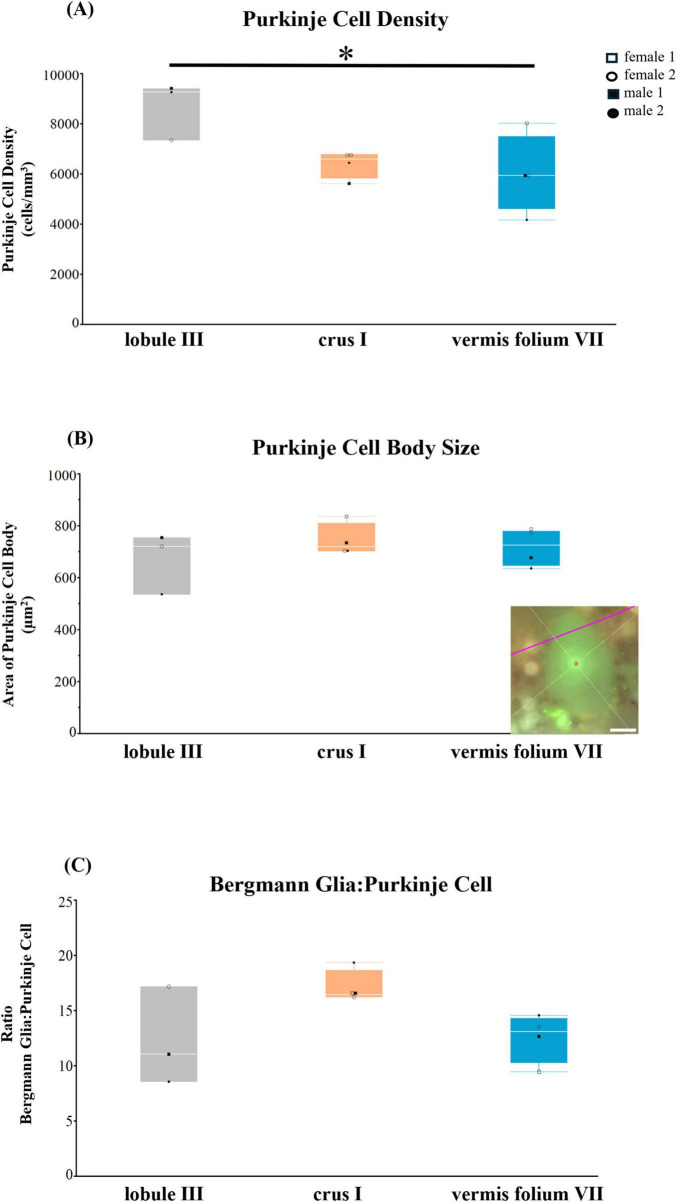
Purkinje cell parameters in the human cerebellum. **(A)** Purkinje cell density (cells/mm^3^). Vermis folium VII had lower PC densities compared to lobule III (31%, *p* = 0.0426) **(B)** Purkinje cell body size (μm^2^) did not differ across lobules. **(C)** Ratio of Bergmann glia (BG) to one Purkinje cell (PC). Crus I lobule had the highest ratio with 17 BG:1 PC compared to lobule III and vermis (12 BG:1 PC), however this was not significant. Representative example of the nucleator probe emitting four rays to measure Purkinje cell body size, inset B. Image acquired on a Zeiss ApoTome2 Axio Imager.M2 microscope system, 63×. Scale bar 10 μm. *p < 0.05.

With the knowledge that PCs are the main inhibitory output neurons of the cerebellar cortex that are tightly connected with BG (De Zeeuw and Hoogland, 2015), we next sought to quantify PCs in lobule III, crus I, and in the vermis lobule VIIA folium in the human cerebellum (Figure 7). Age was positively correlated with PC cell body size (Spearman ρ = 0.6715, *p* = 0.0237) while pH was negatively correlated with PC cell body size (Spearman ρ = −0.6139, *p* = 0.0445). PC densities differed across cerebellar lobules [*F*(2,8) = 4.9340, *p* = 0.0402], with vermis lobule VIIA, folium having significantly lower densities than lobule III (31%, *p* = 0.0426) (Figure 7A). PC body size did not differ across cerebellar lobules including age and pH in the model *F*(4,6.0) = 3.2905, *p* = 0.0940 (Figure 7B). The number of BG surrounding 1 PC also did not significantly differ across cerebellar lobules analyzed [*F*(2,8) = 3.7116, *p* = 0.0724], although the highest ratio was observed in crus I lobule with 17 BG:1 PC compared to lobule III and vermis where approximately 12 BG surrounded 1 PC (Figure 7C).

Overall, we observed subtle differences in PC parameters when comparing lobule III, crus I, and vermis lobule VIIA, folium in the human cerebellum. However, similar to ALDH1L1+ astrocyte densities, the vermis lobule VIIA, folium displayed the lowest PC densities.

### 3.6 Cross-species comparison with human cerebellar astrocytes and Purkinje cells

**FIGURE 8:**
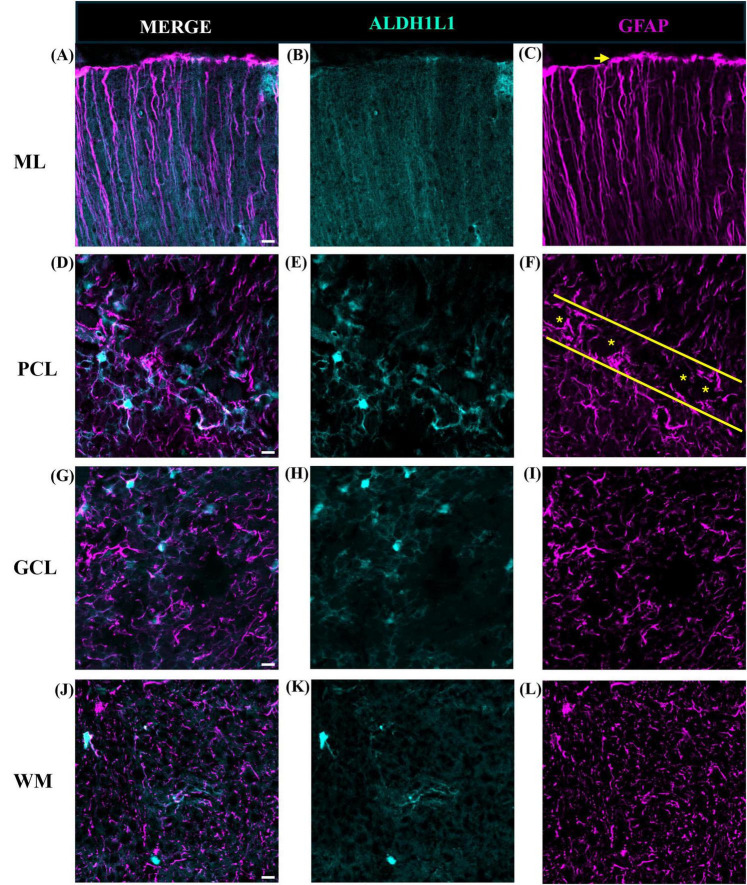
Representative images of astrocytes in the mouse cerebellum. **(A–C)** Molecular layer (ML), **(D–F)** Purkinje cell layer (PCL), **(G–I)** granule cell layer (GCL), and **(J–L)** white matter (WM). In the ML, GFAP-IR processes were less extensive displaying few branched and horizontal processes **(C)**. Bulbous astrocytic end feet were observed at the upper limit of the ML (**C**, yellow arrow). In the PCL (demarked by yellow solid outline, **F**), GFAP-IR processes were prominent with ALDH1L1-IR cell bodies surrounding Purkinje cells (**F**, yellow asterisks). In the GCL astrocyte cell bodies showed robust ALDH1L1-IR labeling **(H)** while GFAP was highly visible in astrocytic processes **(I)**. In the WM, typical fibrous astrocytes were largely GFAP-IR (**K** vs. **L**). tdTomato reporter signal was used to visualize mouse ALDH1L1-IR astrocytes. Images were acquired using a Zeiss ApoTome2 Axio Imager.M2 microscope system, 40×. Scale bar 10 μm. ALDH1L1, aldehyde dehydrogenase-1 family member L1; GFAP, glial fibrillary acidic protein; IR, immunoreactive.

**FIGURE 9:**
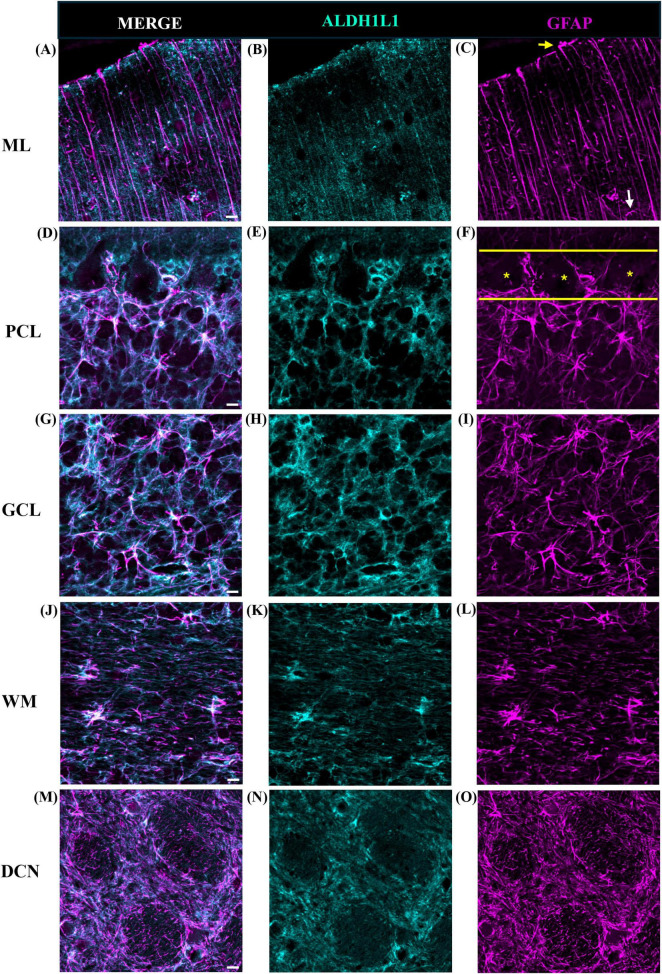
Representative images of astrocytes in the macaque cerebellum. **(A–C)** Molecular layer (ML), **(D–F)** Purkinje cell layer (PCL), **(G–I)** granule cell layer (GCL), **(J–L)** white matter (WM), and **(M–O)** deep cerebellar nuclei (DCN). In the ML, the presence of lateral appendages on Bergmann glia processes were observed (**C**, white arrow). Similar to mice, bulbous astrocytic end feet were observed at the upper limit (**C**, yellow arrow). In the PCL (demarked by yellow solid outline, **F**), GFAP-IR processes were prominent with ALDH1L1-IR cell bodies surrounding Purkinje cells (**F**, yellow asterisks). In the GCL, astrocytes displayed a mesh-like patterning. In the WM, similar to humans, fibrous astrocytes presented strong labeling for ALDH1L1 **(K)** and GFAP **(L)**. In the DCN, fastigial nucleus is shown, ALDH1L1-IR **(N)** and GFAP-IR **(O)** astrocyte cell bodies and processes showed a discrete organization. Images were acquired using a Zeiss ApoTome2 Axio Imager.M2 microscope system, 40×, scale bar 10 μm **(A–L)**, 20×, scale bar 20 μm **(M–O)**. ALDH1L1, aldehyde dehydrogenase-1 family member L1; GFAP, glial fibrillary acidic protein; IR, immunoreactive.

**FIGURE 10:**
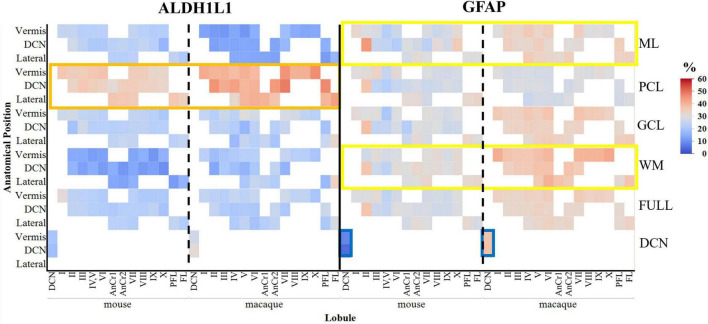
Area coverage of ALDH1L1-IR and GFAP-IR astrocytes in cerebellar layers and lobules in mice and macaques (percentages are reported). Note the high expression of GFAP-IR astrocytes in the molecular layer and in the white matter compared to ALDH1L1-IR astrocytes (yellow rectangles). Similar to the human cerebellum, ALDH1L1-IR astrocytes were highly expressed in the Purkinje cell layer in macaques and a lesser extent in mice (orange rectangle). However, the % area coverage of ALDH1L1-IR astrocytes in the PCL were considerably lower in mice (35%) and macaques (41%) compared to humans (53%). GFAP-IR astrocytes in the deep cerebellar nuclei in mice displayed very low coverage compared to macaques (blue rectangle) and humans (compare with Figure 4). White areas indicate unavailable data due to these lobules not being present in the sections analyzed. tdTomato reporter signal was used to visualize mouse ALDH1L1-IR astrocytes. Full indicates average of all cerebellar layers excluding the DCN. ALDH1L1, aldehyde dehydrogenase-1 family member L1; DCN, deep cerebellar nuclei; GCL, granule cell layer; GFAP, glial fibrillary acidic protein; IR, immunoreactive; ML, molecular layer; PCL, Purkinje cell layer; WM, white matter.

**FIGURE 11:**
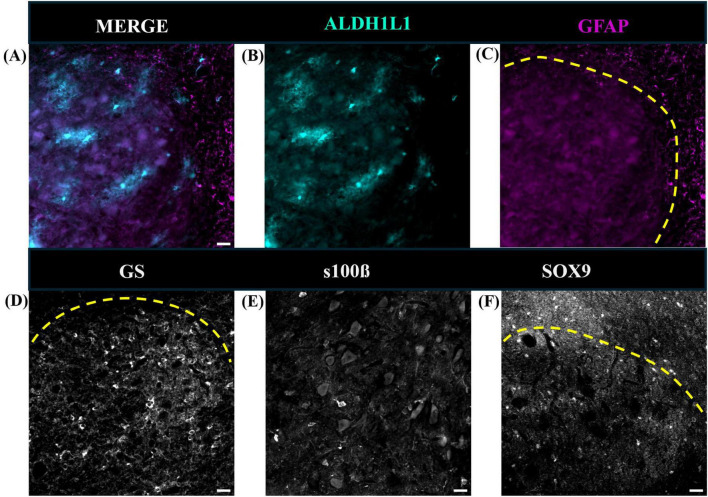
Representative images of cerebellar astrocytes in the mouse deep cerebellar nuclei. Fastigial nucleus is shown. While ALDH1L1-IR astrocyte cell bodies and processes were present **(A,B)**, GFAP-IR astrocytes were scarce **(C)**. Additional immunolabeling revealed that glutamine synthetase **(D)**, S100β **(E)**, and sox9 **(F)** positive astrocytes were present in the mouse DCN. Yellow dashed line outlines DCN in panels **(C,D,F)**. tdTomato reporter signal was used to visualize mouse ALDH1L1-IR astrocytes. Images were acquired using a Zeiss ApoTome2 Axio Imager.M2 microscope system, 20×. Scale bar 20 μm. ALDH1L1, aldehyde dehydrogenase-1 family member L1; DCN, deep cerebellar nuclei; GFAP, glial fibrillary acidic protein; IR, immunoreactive.

**FIGURE 12:**
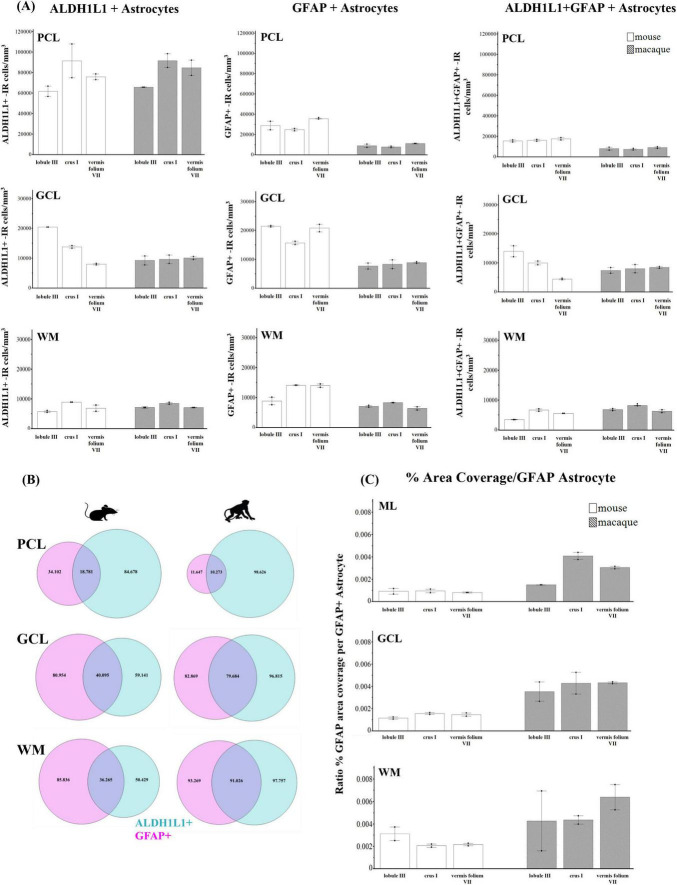
Astrocyte densities in the mouse and macaque cerebellum. **(A)** ALDH1L1+ and GFAP+-IR (immunoreactive) astrocytes, cells/mm^3^, in cerebellar layers and functionally distinct lobules of interest in mice and macaques. In both species, ALDH1L1+-IR astrocytes showed the highest densities in the PCL and the lowest in WM which was similar to humans (compare with Figures 5A–C). Opposing trends were generally observed across species between the astrocytic markers with ALDH1L1+-IR astrocytes increasing with species complexity while GFAP+-IR astrocytes decreased from mice to macaques to humans (compare with Figure 5). **(B)** Proportion of ALDH1L1+ and GFAP+ astrocytes in cerebellar layers across species. For all species, most astrocytes in the PCL were ALDH1L1+ whereas astrocytes in the WM were largely GFAP+. Most astrocytes in the mouse cerebellum were GFAP+. **(C)** GFAP-defined astrocytic territories (percentage per GFAP+ astrocyte) increased from mice to macaques to humans across cerebellar layers (compare with Figure 5E). tdTomato reporter signal was used to visualize mouse ALDH1L1-IR astrocytes. ALDH1L1, aldehyde dehydrogenase-1 family member L1; GCL, granule cell layer; GFAP, glial fibrillary acidic protein; ML, molecular layer; PCL, Purkinje cell layer; WM, white matter.

**FIGURE 13:**
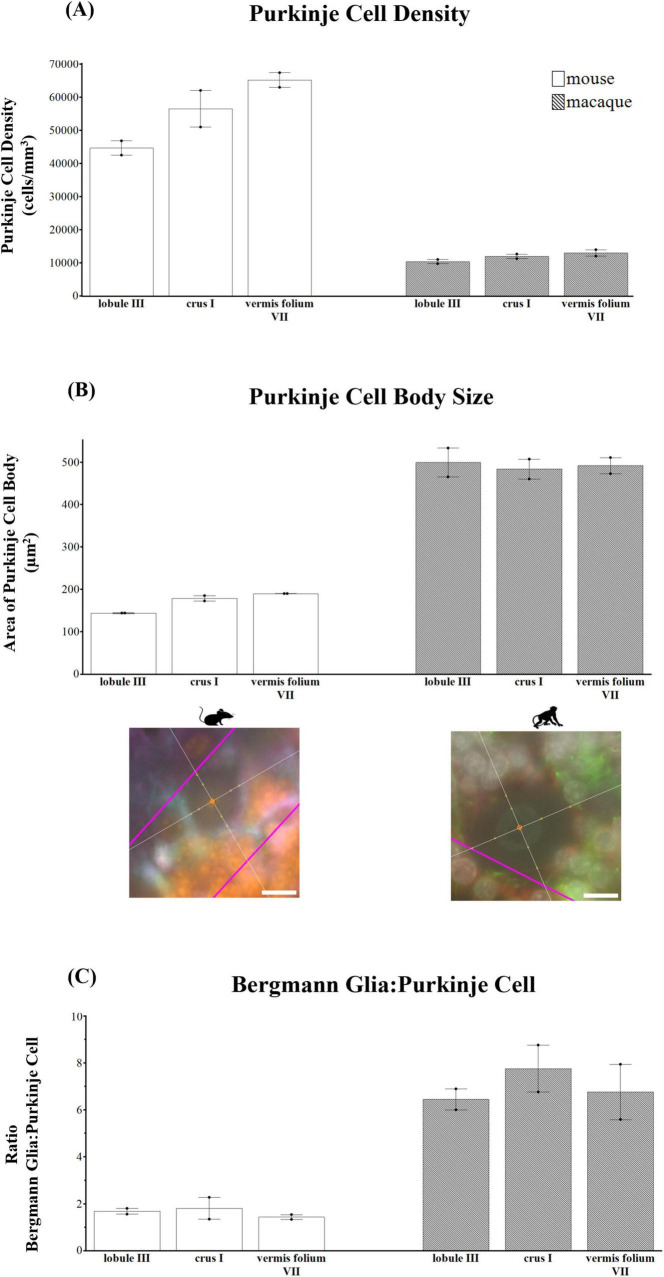
Purkinje cell parameters in mice and macaques. **(A)** Purkinje cell density (cells/mm^3^). Purkinje cell densities decreased with species evolution (approximately 2× lower in humans compared to macaques and 8× lower than mice, compare Figure 6A with panel A). **(B)** Purkinje cell body area (μm^2^). Purkinje cell body sizes increased from mice to macaques to humans (compare Figure 6B with panel B). Representative examples of the nucleator probe emitting four rays to measure Purkinje cell body size are shown for mouse and macaque. **(C)** Ratio of Bergmann glia (BG) to one Purkinje cell (PC) showing that the number of BG surrounding each PC increased from mice to macaques to humans where humans displayed an average of 14 BG:1 PC compared to 7 BG:1 PC in macaques and 2 BG:1 PC in mice (compare Figure 6C with panel C). tdTomato reporter signal was used to visualize mouse ALDH1L1-IR astrocytes. Images acquired on a Zeiss ApoTome2 Axio Imager.M2 microscope system, 63×. Scale bar 10 μm.

Comparative neuroanatomical studies have highlighted astrocyte diversity across species in the cerebral cortex (Colombo and Reisin, 2004; Oberheim et al., 2006, 2009; O’Leary et al., 2020), however, whether species-specific features and distributions of astrocytic subtypes exist in hindbrain regions such as the cerebellum have not been well studied. Qualitative comparisons of cerebellar astrocytes revealed that the complexity of ML processes increased from mice to macaques to humans, with humans displaying more branched and horizontal processes. We did not observe varicosities or knotted blebbings in mice and macaques (compare Figures 3, 8A–C, 9A–C). In mice and macaques, GFAP-IR bulbous endfeet were strikingly visible near the upper limit of the ML (Figures 8C, 9C). Similar to humans, ALDH1L1-IR cell bodies were dominant in the PCL and were in close proximity to PCs in macaques and lesser extent in mice (Figures 8D–F, 9D–F). However, the % area coverage of ALDH1L1-IR astrocytes in the PCL were considerably lower in mice (35%) and macaques (41%) compared to humans (53%) (compare Figures 5, 10). Astrocytes in the GCL (Figures 8G–I, 9G–I) and WM (Figures 8J–L, 9J–L) were visible in mice and macaques, with the latter being highly GFAP-IR, similar to human WM astrocytes (compare Figures 4G–I with 8J–L, 9J–L). Nonetheless, the % coverages of GFAP-IR astrocytes in WM were lower in mice (30%) and macaques (38%) compared to humans (43%) (compare Figures 5, 10). Similarly, ALDH1L1-IR astrocyte % coverages in mice were strikingly low (13%) compared to macaques (21%) and humans (30%) (compare Figures 5, 10). Within the DCN, we observed robust species differences in astrocytes. Qualitatively, while ALDH1L1-IR astrocytes were in high abundance in the DCN of mice (Figures 11A, B), there was a dramatic absence of GFAP-IR astrocytes (Figures 11A, C). Further immunolabeling of glutamine synthetase (Figure 11D), S100 (Figure 11E), and sox9 (Figure 11F), confirmed the presence of astrocytes in mouse DCN. In macaque DCN, astrocyte processes were highly visible and appeared discretely organized while astrocyte cell bodies were difficult to distinguish from processes (Figures 9M–O). Complementing such observations, area coverages of GFAP-IR astrocytes in the DCN were strikingly lower in mice (6%) compared to macaques (38%) and humans (41%) (compare Figure 5, 10). We next generated stereological estimates of ALDH1L1 and GFAP astrocytes in three functionally distinct lobules of interest (lobule III, crus I, and vermis lobule VIIA, folium) in each cerebellar layer (Figure 12). We observed opposing trends with ALDH1L1 densities increasing with species evolution, approximately 1× higher in humans compared to macaques and mice, while GFAP densities decreased from mice to macaques to humans, 1–2× lower in humans than macaques and 2–5× lower compared to mice depending on the layer of interest (compare Figures 6A–C, 12A). Furthermore, most astrocytes in the mouse cerebellum were GFAP (Figure 12B). Profiting from our astrocytic % area coverages and densities, we estimated GFAP-defined territories in mice and macaques as previously demonstrated in our human analysis. In general, we observed larger GFAP-defined territories in humans compared to mice and macaques across all cerebellar layers which is consistent with previous descriptions of cerebral astrocytes (Oberheim et al., 2006, 2009; O’Leary et al., 2020) (compare Figure 6E, 12C). Notably, the ML (presumed to contain largely BG processes) displayed the largest discrepancies with human GFAP territories being 3× larger than macaques and 10× larger than mice. Lastly, we quantified PC parameters in mice and macaques to compare with our human PC data (compare Figure 7, 13). We observed that while PC densities decreased with species evolution (approximately 2× lower in humans compared to macaques and 8× lower than mice, Figures 7A, 13A), cell body sizes increased being 1× larger in humans compared to macaques and 4× larger when comparing to mice (Figures 7B, 13B). Additionally, the number of BG surrounding each PC increased from mice to macaques to humans where humans displayed an average of 14 BG surrounding 1 PC compared to 7 BG to 1 PC in macaques and 2 BG to 1 PC in mice (Figures 7C, 13C).

Overall, these findings align with the growing literature for astrocyte and PC heterogeneity and suggest important species-specific differences of cerebellar astrocytes and PCs.

## 4 Discussion

In this study we performed a comprehensive neuroanatomical investigation to characterize astrocytes and PCs in the healthy human cerebellum. We first visualized astrocytes observing differential immunoreactivity of canonical astrocyte markers, GFAP and ALDH1L1 across the cerebellar layers. These observations were quantitatively confirmed with % area coverages of our astrocyte markers being measured at three anatomical positions within a complete cerebellar hemisphere. Next, robust stereological estimates of astrocytes in three functionally distinct cerebellar lobules revealed (1) the vermis lobule VIIA, folium displayed the lowest densities of ALDH1L1+ astrocytes and (2) the highest ALDH1L1+ densities were found in the PCL while GFAP+ densities and astrocytes colocalizing (ALDH1L1+ GFAP+) were highest in the GCL. Furthermore, by assessing GFAP-defined territories we observed that astrocytes in the GCL displayed the smallest territories while those in the ML and WM had among the largest territories in the human cerebellum. Additionally, we performed an in-depth analysis of PCs. While subtle differences in PC parameters were observed, vermis lobule VIIA, folium showed the lowest PC densities while there was a trend toward the cognitive lobule, crus I, having the highest BG:PC ratio. Finally, we compared human cerebellar astrocyte and PC observations with those of animals often used to model human illnesses, namely mice and macaques, highlighting important species-specific differences and similarities.

Our qualitative observations indicate that multiple astrocyte markers are needed to capture the heterogeneity of cerebellar astrocytes across layers. We highlighted ALDH1L1 as a suitable and robust marker for BG while GFAP was prominent in BG processes and cerebellar fibrous astrocytes in the human cerebellum. Varicosity-like protrusions and knotted blebbings on GFAP-IR BG processes were observed exclusively in human postmortem tissues. Further analyses are needed to determine if these features are present in all individuals or if they are solely observed under specific conditions as has been previously reported (Falcone et al., 2022). There is the possibility that such astrocytic features are the result of confounding variables in humans such as longer PMIs. However, astrocytic varicosities were observed from human tissues obtained from surgical resections (Oberheim et al., 2009; Sosunov et al., 2014). Additionally, in a recent rigorous comparative study, varicosities were also observed in ape species with PMIs less than 24 h with no significant correlations between the presence of varicosities in humans and apes and PMI, age, cause of death, nor with microglia cell number and activation (Falcone et al., 2022). It remains unclear if there exists a functional relevance to these varicosities; ultrastructural imaging techniques may aid toward this understanding. A striking observation was the scarcity of GFAP+ astrocytes in the DCN of mice, which has been previously reported (Shibuki et al., 1996). This observation appeared specific to the marker GFAP as ALDH1L1, glutamine synthetase, S100β, and sox9 were all visualized in the DCN, suggesting limited intermediate filaments in DCN astrocytes in healthy mice. An absence of intermediate filaments in astrocytes was reported to have no major consequences on non-reactive astrocytes, perhaps due to other cytoskeleton components, such as actin filaments compensating to support the development and maintenance of astrocytic processes (Wilhelmsson et al., 2004).

Mapping ALDH1L1-IR and GFAP-IR % area coverages of astrocytes across three anatomical positions (lateral, DCN, and vermis) within a complete cerebellar hemisphere, indicated that astrocytes are indeed present across all lobules showing subtle differences. However, we consistently observed higher ALDH1L1 coverages and densities in the PCL. Such results could suggest a higher metabolic demand needed in BG as ALDH1L1 is a key enzyme in folate metabolism which is important in nucleotide biosynthesis and cell division (Krupenko, 2009; Yang et al., 2011). Indeed, BG processes are highly dynamic within the ML, interacting with both excitatory and inhibitory synapses making them crucial in the regulation of cerebellum synaptic transmission (Buffo and Rossi, 2013; De Zeeuw and Hoogland, 2015). A higher metabolic demand might also be necessary in human astrocytes and would aid in explaining the increased ALDH1L1+ densities with species evolution. In support, Zhang et al. (2016) showed an upregulation of genes involved in different aspects of metabolism in humans compared to mouse astrocytes.

With respect to GFAP, we first observed a positive correlation between % area coverages of GFAP-IR astrocytes and age which strengthens our confidence in these estimations as reports suggest that aging is often accompanied by the upregulation of GFAP (Wruck and Adjaye, 2020; Popov et al., 2023). GFAP coverages were abundant in BG processes which often displayed complex and lateral appendages in humans and to a lesser extent in macaques. Such horizontal branching’s have been previously observed in marmosets (Muñoz et al., 2021). GFAP was also enhanced in WM cerebellar astrocytes suggesting similarities to cerebral fibrous astrocytes with an increased propensity for intermediate filament proteins in this astrocytic subtype (Oberheim et al., 2009). Indeed, a recent single cell-RNA sequencing and spatial transcriptomics study in the mouse brain, found GFAP to be among the top enriched genes in WM astrocytes along with an enrichment for genes involved in cytoskeletal regulation such as Marcks, Marcks11, and Lima1 likely influencing their distinct morphology (Bocchi et al., 2025). Importantly, while molecular commonalities were observed between WM cerebellar astrocytes and cerebral WM astrocytes, distinct molecular features were also reported further highlighting the molecular and functional diversity found both within astrocytic subtypes and across brain regions (Morel et al., 2017; Batiuk et al., 2020; Bocchi et al., 2025). In contrast to ALDH1L1+ densities increasing with species evolution, human astrocytes displayed 2–5× lower GFAP+ densities than mice depending on the layer of interest. While somewhat unexpected, past studies have reported an inverse relationship between GFAP and evolution (Bodega et al., 1994; Kálmán, 2002). GFAP is an intermediate filament protein involved with structural stabilization of astrocyte processes (Yang and Wang, 2015) and thus might be expected to increase with the complexity of processes. However, Viana et al. (2023) recently reported in mice, that while the hippocampal layers, CA1 radiatum and DG molecular, showed the lowest astrocyte densities, these layers compensated by displaying the largest defined territories. In support, while we observed the highest GFAP+ astrocyte densities in the GCL, these astrocytes displayed the smallest territories, in the human cerebellum. Similarly, GFAP+ astrocyte territories were among the largest in the ML and WM, with the latter displaying the lower GFAP+ astrocyte densities. Further aligning with these observations, our cross-species analyses showed lower GFAP+ astrocyte densities in the human cerebella, compared to mice and macaques, yet the territories of human astrocytes were 3–10× larger depending on the layer the astrocytes were located in. This is consistent with cerebral astrocytes displaying increased complexity in the human brain (Oberheim et al., 2006, 2009; O’Leary et al., 2020).

Finally, we analyzed PCs as these neurons are highly interconnected with BG (Yamada et al., 2000; Bellamy, 2006; Miyazaki et al., 2017). While subtle differences in PC parameters were observed when comparing three functionally distinct lobules (lobule III, crus I, and vermis lobule VIIA, folium) in the human cerebellum, our cross-species analyses highlighted important species differences. Previous reports have characterized PCs across species and, in accordance with the present study, PC density decreases accompanied by significant increases in cell size and spacing of PCs have been described with increasing evolution (Lange, 1975; Korbo and Andersen, 1995). A recent study elegantly compared PCs in humans and mice, finding that human PCs display higher dendritic complexities and present 7.5× more dendritic spines along dendrites, processing 6.5× more input patterns than in mice PCs, suggesting greater computational capabilities in humans (Masoli et al., 2024). Interestingly, with evolution, we observed an increase in the number of BG surrounding one PC with the highest ratio being in crus I compared to lobule III and vermis lobule VIIA, folium. Considering crus I is involved in cognitive processing, our results may thus be indicative of an increased need for BG in higher level cognitive cerebellar functioning, suggesting greater computational processing in the human cerebellum (Masoli et al., 2024).

Few studies have interrogated lobule and layer specificities of PC and astrocytic distributions. Kozareva et al. (2021) used transcriptional profiling to characterize PC diversity across 16 lobules in the mouse cerebellum. They found that PCs displayed considerable heterogeneity, particularly in posterior lobules, uvula (lobule IX) and nodulus (lobule X). The authors also report regional specialization of BG in lobule VI, the uvula (lobule IX) and nodulus (lobule X). Although we did not perform PC analyses in these lobules, it is interesting to note that crus I (also a posterior lobule) displayed higher BG to PC ratios compared to lobule III (anterior lobule) and to the vermis lobule in humans. Such findings together with our observations highlight the importance of performing detailed lobule and layer assessments to parcellate the cellular heterogeneity observed within the cerebellum.

This study has several important limitations. First, we recognize that our sample sizes were limited. While we included male and female human individuals, increasing our sample would allow us to confidently disentangle potential sex differences in PC and astrocytes within the cerebellum. Similarly, while our human postmortem comparisons to those of mice and macaques provide initial insights, they should be interpreted as preliminary given the low sample size which precluded statistical analyses. Future studies should include larger samples sizes to validate these findings and further explore species-specific observations. Second, while we attempted to define GFAP territories, the 2D method we used was rudimentary as we did not perform an in-depth Sholl analysis which would have allowed for 3D representations. However, labeling astrocytes in their entirety in the postmortem human brain is challenging. While vimentin is a promising marker for visualizing the complexities of astrocytes, we observed limited labeling in the cerebellum (1–2 vimentin+ astrocytes/section in humans, unpublished data). Such observations are in line with those reported in other subcortical structures such as the thalamus, where minimal vimentin+ astrocytes have been observed (O’Leary et al., 2020). Finally, when interpreting our cross-species analyses, caution needs to be taken when comparing ALDH1L1+ astrocytes in mice, as the transgenic Aldh1L1-Cre/ERT2; Rosa26-TdTomato mice displayed a more complete staining pattern with intense labeling of cell bodies and astrocyte processes that likely skewed the % area coverages when compared to antibody labeling. Intuitively, labeling human and macaque astrocytes in their entirety would reveal even greater species differences and thus our results likely reflect conservative estimates in these species. Follow-up investigations would gain from comparing the mRNA expression of GFAP and ALDH1L1 astrocytic markers.

Despite these limitations, we systematically examined cerebellar astrocytes and PCs in the healthy human cerebellum which extends the growing literature of astrocyte heterogeneity to the cerebellum. Furthermore, we highlight species divergence in PC and astrocytic features important for our understanding of animal models of disease and translation of findings from these species to human patients. This study is timely considering the increasing evidence for the cerebellum in higher order cognitive and emotional brain functioning (Buckner, 2013; Fastenrath et al., 2022) where growing literature demonstrates aberrant structure and function in neurodevelopmental (Haldipur et al., 2022; Hwang et al., 2022) and psychiatric disorders (Depping et al., 2018; Romer et al., 2018; Chambers et al., 2022). Thus, this study provides foundational knowledge for cerebellar astrocyte and PC properties unique to the healthy brain, allowing future directions to next determine dysfunction of these properties in central nervous system disease and other disorders.

## Data Availability

The raw data supporting the conclusions of this article will be made available by the authors, without undue reservation.

## Footnotes

1
https://douglasbrainbank.ca

## References

[B1] Allen Institute (2004). *Allen mouse brain atlas.* Seattle, WA: Allen Institute.

[B2] Alvarez J. I. Katayama T. Prat A. (2013). Glial influence on the blood brain barrier. *Glia* 61 1939–1958. 10.1002/glia.22575 24123158 PMC4068281

[B3] Bankhead P. Loughrey M. B. Fernández J. A. Dombrowski Y. McArt D. G. Dunne P. D. (2017). QuPath: Open source software for digital pathology image analysis. *Sci. Rep.* 7:16878. 10.1038/s41598-017-17204-5 29203879 PMC5715110

[B4] Barton R. A. Venditti C. (2014). Rapid evolution of the cerebellum in humans and other great apes. *Curr. Biol.* 24 2440–2444. 10.1016/j.cub.2014.08.056 25283776

[B5] Batiuk M. Y. Martirosyan A. Wahis J. de Vin F. Marneffe C. Kusserow C. (2020). Identification of region-specific astrocyte subtypes at single cell resolution. *Nat. Commun.* 11:1220. 10.1038/s41467-019-14198-8 32139688 PMC7058027

[B6] Bayraktar O. A. Bartels T. Holmqvist S. Kleshchevnikov V. Martirosyan A. Polioudakis D. (2020). Astrocyte layers in the mammalian cerebral cortex revealed by a single-cell in situ transcriptomic map. *Nat. Neurosci.* 23 500–509. 32203496 10.1038/s41593-020-0602-1 PMC7116562

[B7] Bellamy T. C. (2006). Interactions between Purkinje neurones and Bergmann glia. *Cerebellum* 5 116–126. 10.1080/14734220600724569 16818386

[B8] Bocchi R. Thorwirth M. Simon-Ebert T. Koupourtidou C. Clavreul S. Kolf K. (2025). Astrocyte heterogeneity reveals region-specific astrogenesis in the white matter. *Nat. Neurosci.* 28 457–469. 10.1038/s41593-025-01878-6 39994409 PMC11893471

[B9] Bodega G. Suárez I. Rubio M. Fernández B. (1994). Ependyma: Phylogenetic evolution of glial fibrillary acidic protein (GFAP) and vimentin expression in vertebrate spinal cord. *Histochemistry* 102 113–122. 10.1007/BF00269015 7822213

[B10] Buckner R. L. (2013). The cerebellum and cognitive function: 25 years of insight from anatomy and neuroimaging. *Neuron* 80 807–815. 10.1016/j.neuron.2013.10.044 24183029

[B11] Buckner R. L. Krienen F. M. Castellanos A. Diaz J. C. Yeo B. T. (2011). The organization of the human cerebellum estimated by intrinsic functional connectivity. *J. Neurophysiol.* 106 2322–2345. 10.1152/jn.00339.2011 21795627 PMC3214121

[B12] Buffo A. Rossi F. (2013). Origin, lineage and function of cerebellar glia. *Prog. Neurobiol.* 109 42–63. 10.1016/j.pneurobio.2013.08.001 23981535

[B13] Cerrato V. (2020). Cerebellar astrocytes: Much more than passive bystanders in ataxia pathophysiology. *J. Clin. Med.* 9:757. 10.3390/jcm9030757 32168822 PMC7141261

[B14] Cerrato V. Parmigiani E. Figueres-Oñate M. Betizeau M. Aprato J. Nanavaty I. (2018). Multiple origins and modularity in the spatiotemporal emergence of cerebellar astrocyte heterogeneity. *PLoS Biol.* 16:e2005513. 30260948 10.1371/journal.pbio.2005513 PMC6178385

[B15] Chambers T. Escott-Price V. Legge S. Baker E. Singh K. D. Walters J. T. R. (2022). Genetic common variants associated with cerebellar volume and their overlap with mental disorders: A study on 33,265 individuals from the UK-Biobank. *Mol. Psychiatry* 27 2282–2290. 10.1038/s41380-022-01443-8 35079123 PMC9126806

[B16] Chung W.-S. Allen N. J. Eroglu C. (2015). Astrocytes control synapse formation, function, and elimination. *Cold Spring Harb. Perspect. Biol.* 7:a020370. 10.1101/cshperspect.a020370 25663667 PMC4527946

[B17] Colombo J. A. Reisin H. D. (2004). Interlaminar astroglia of the cerebral cortex: A marker of the primate brain. *Brain Res.* 1006 126–131. 10.1016/j.brainres.2004.02.003 15047031

[B18] De Zeeuw C. I. Hoogland T. M. (2015). Reappraisal of Bergmann glial cells as modulators of cerebellar circuit function. *Front. Cell. Neurosci.* 9:246. 10.3389/fncel.2015.00246 26190972 PMC4488625

[B19] Depping M. S. Schmitgen M. M. Kubera K. M. Wolf R. C. (2018). Cerebellar contributions to major depression. *Front. Psychiatry* 9:634. 10.3389/fpsyt.2018.00634 30555360 PMC6281716

[B20] Díaz-Castro B. Robel S. Mishra A. (2023). Astrocyte endfeet in brain function and pathology: Open questions. *Annu. Rev. Neurosci.* 46 101–121. 10.1146/annurev-neuro-091922-031205 36854317

[B21] Endo F. Kasai A. Soto J. S. Yu X. Qu Z. Hashimoto H. (2022). Molecular basis of astrocyte diversity and morphology across the CNS in health and disease. *Science* 378:eadc9020. 10.1126/science.adc9020 36378959 PMC9873482

[B22] Falcone C. McBride E. L. Hopkins W. D. Hof P. R. Manger P. R. Sherwood C. C. (2022). Redefining varicose projection astrocytes in primates. *Glia* 70 145–154. 10.1002/glia.24093 34533866

[B23] Falcone C. Wolf-Ochoa M. Amina S. Hong T. Vakilzadeh G. Hopkins W. D. (2019). Cortical interlaminar astrocytes across the therian mammal radiation. *J. Comp. Neurol.* 527 1654–1674. 10.1002/cne.24605 30552685 PMC6465161

[B24] Fastenrath M. Spalek K. Coynel D. Loos E. Milnik A. Egli T. (2022). Human cerebellum and corticocerebellar connections involved in emotional memory enhancement. *Proc. Natl. Acad. Sci. U.S.A.* 119:e2204900119. 10.1073/pnas.2204900119 36191198 PMC9564100

[B25] Forrest S. L. Kim J. H. Crockford D. R. Huynh K. Cheong R. Knott S. (2023). Distribution patterns of astrocyte populations in the human cortex. *Neurochem. Res.* 48 1222–1232. 10.1007/s11064-022-03700-2 35930103 PMC10030423

[B26] Goertzen A. Veh R. W. (2018). Fañanas cells-the forgotten cerebellar glia cell type: Immunocytochemistry reveals two potassium channel-related polypeptides, Kv2.2 and Calsenilin (KChIP3) as potential marker proteins. *Glia* 66 2200–2208. 10.1002/glia.23478 30151916

[B27] Haldipur P. Millen K. J. Aldinger K. A. (2022). Human Cerebellar Development and Transcriptomics: Implications for Neurodevelopmental Disorders. *Annu. Rev. Neurosci.* 45 515–531. 10.1146/annurev-neuro-111020-091953 35440142 PMC9271632

[B28] Hoogland T. M. Kuhn B. (2010). Recent developments in the understanding of astrocyte function in the cerebellum in vivo. *Cerebellum* 9 264–271. 10.1007/s12311-009-0139-z 19904577

[B29] Hwang I. Kim B. S. Ko H. R. Cho S. Lee H. Y. Cho S. W. (2022). Cerebellar dysfunction and schizophrenia-like behavior in Ebp1-deficient mice. *Mol. Psychiatry* 27 2030–2041. 10.1038/s41380-022-01458-1 35165395

[B30] Kálmán M. (2002). GFAP expression withdraws–a trend of glial evolution? *Brain Res. Bull.* 57 509–511. 10.1016/s0361-9230(01)00713-4 11923020

[B31] Karpf J. Unichenko P. Chalmers N. Beyer F. Wittmann M. T. Schneider J. (2022). Dentate gyrus astrocytes exhibit layer-specific molecular, morphological and physiological features. *Nat. Neurosci.* 25 1626–1638. 10.1038/s41593-022-01192-5 36443610

[B32] Kawabata K. Bagarinao E. Watanabe H. Maesawa S. Mori D. Hara K. (2022). Functional connector hubs in the cerebellum. *Neuroimage* 257:119263. 10.1016/j.neuroimage.2022.119263 35500805

[B33] Klein A. P. Ulmer J. L. Quinet S. A. Mathews V. Mark L. P. (2016). Nonmotor functions of the cerebellum: An introduction. *Am. J. Neuroradiol.* 37 1005–1009. 10.3174/ajnr.A4720 26939633 PMC7963530

[B34] Korbo L. Andersen B. B. (1995). The distributions of Purkinje cell perikaryon and nuclear volume in human and rat cerebellum with the nucleator method. *Neuroscience* 69 151–158. 10.1016/0306-4522(95)00223-6 8637613

[B35] Kozareva V. Martin C. Osorno T. Rudolph S. Guo C. Vanderburg C. (2021). A transcriptomic atlas of mouse cerebellar cortex comprehensively defines cell types. *Nature* 598 214–219. 10.1038/s41586-021-03220-z 34616064 PMC8494635

[B36] Kreutz A. Barger N. (2018). Maximizing explanatory power in stereological data collection: A protocol for reliably integrating optical fractionator and multiple immunofluorescence techniques. *Front. Neuroanat.* 12:73. 10.3389/fnana.2018.00073 30425623 PMC6218486

[B37] Krupenko S. A. (2009). FDH: An aldehyde dehydrogenase fusion enzyme in folate metabolism. *Chem. Biol. Interact.* 178 84–93. 10.1016/j.cbi.2008.09.007 18848533 PMC2664990

[B38] Lange W. (1975). Cell number and cell density in the cerebellar cortex of man and some other mammals. *Cell Tissue Res.* 157 115–124. 10.1007/BF00223234 804353

[B39] Madigan J. C. Carpenter M. B. (1971). *Cerebellum of the rhesus monkey: Atlas of lobules, laminae, and folia, in sections.* University Park, PA: University Park Press.

[B40] Madisen L. Zwingman T. A. Sunkin S. M. Oh S. W. Zariwala H. A. Gu H. (2010). A robust and high-throughput Cre reporting and characterization system for the whole mouse brain. *Nat. Neurosci.* 13 133–140. 10.1038/nn.2467 20023653 PMC2840225

[B41] Masoli S. Sanchez-Ponce D. Vrieler N. Abu-Haya K. Lerner V. Shahar T. (2024). Human Purkinje cells outperform mouse Purkinje cells in dendritic complexity and computational capacity. *Commun. Biol.* 7:5. 10.1038/s42003-023-05689-y 38168772 PMC10761885

[B42] Matias I. Morgado J. Gomes F. C. A. (2019). Astrocyte heterogeneity: Impact to brain aging and disease. *Front. Aging Neurosci.* 11:59. 10.3389/fnagi.2019.00059 30941031 PMC6433753

[B43] Mayorquin L. C. Rodriguez A. V. Sutachan J. J. Albarracín S. L. (2018). Connexin-mediated functional and metabolic coupling between astrocytes and neurons. *Front. Mol. Neurosci.* 11:118. 10.3389/fnmol.2018.00118 29695954 PMC5905222

[B44] Miyazaki T. Yamasaki M. Hashimoto K. Kohda K. Yuzaki M. Shimamoto K. (2017). Glutamate transporter GLAST controls synaptic wrapping by Bergmann glia and ensures proper wiring of Purkinje cells. *Proc. Natl. Acad. Sci. U.S.A.* 114 7438–7443. 10.1073/pnas.1617330114 28655840 PMC5514701

[B45] Morel L. Chiang M. S. R. Higashimori H. Shoneye T. Iyer L. K. Yelick J. (2017). Molecular and functional properties of regional astrocytes in the adult brain. *J. Neurosci.* 37 8706–8717. 10.1523/JNEUROSCI.3956-16.2017 28821665 PMC5588463

[B46] Mouton P. R. (2002). *Principles and practices of unbiased stereology.* Baltimore, MD: The Johns Hopkins University Press.

[B47] Muñoz Y. Cuevas-Pacheco F. Quesseveur G. Murai K. K. (2021). Light microscopic and heterogeneity analysis of astrocytes in the common marmoset brain. *J. Neurosci. Res.* 99 3121–3147. 10.1002/jnr.24967 34716617 PMC9541330

[B48] Oberheim N. A. Takano T. Han X. He W. Lin J. H. Wang F. (2009). Uniquely hominid features of adult human astrocytes. *J. Neurosci.* 29 3276–3287. 10.1523/JNEUROSCI.4707-08.2009 19279265 PMC2819812

[B49] Oberheim N. A. Wang X. Goldman S. Nedergaard M. (2006). Astrocytic complexity distinguishes the human brain. *Trends Neurosci.* 29 547–553. 10.1016/j.tins.2006.08.004 16938356

[B50] O’Leary L. A. Davoli M. A. Belliveau C. Tanti A. Ma J. C. Farmer W. T. (2020). Characterization of vimentin-immunoreactive astrocytes in the human brain. *Front. Neuroanat.* 14:31. 10.3389/fnana.2020.00031 32848635 PMC7406576

[B51] Popov A. Brazhe N. Morozova K. Yashin K. Bychkov M. Nosova O. (2023). Mitochondrial malfunction and atrophy of astrocytes in the aged human cerebral cortex. *Nat. Commun.* 14:8380. 10.1038/s41467-023-44192-0 38104196 PMC10725430

[B52] Romer A. L. Knodt A. R. Houts R. Brigidi B. D. Moffitt T. E. Caspi A. (2018). Structural alterations within cerebellar circuitry are associated with general liability for common mental disorders. *Mol. Psychiatry* 23 1084–1090. 10.1038/mp.2017.57 28397842 PMC5636639

[B53] Schmahmann J. D. (2019). The cerebellum and cognition. *Neurosci. Lett.* 688 62–75. 10.1016/j.neulet.2018.07.005 29997061

[B54] Schmahmann J. D. Doyon J. Toga A. W. Petrides M. Evans A. C. (2000). *MRI atlas of the human cerebellum.* Cambridge, MA: Academic Press. 10.1006/nimg.1999.0459 10458940

[B55] Schmahmann J. D. Guell X. Stoodley C. J. Halko M. A. (2019). The theory and neuroscience of cerebellar cognition. *Annu. Rev. Neurosci.* 42, 337–364. 10.1146/annurev-neuro-070918-050258 30939101

[B56] Sereno M. I. Diedrichsen J. Tachrount M. Testa-Silva G. d’Arceuil H. De Zeeuw C. (2020). The human cerebellum has almost 80% of the surface area of the neocortex. *Proc. Natl. Acad. Sci. U.S.A.* 117 19538–19543. 10.1073/pnas.2002896117 32723827 PMC7431020

[B57] Shibuki K. Gomi H. Chen L. Bao S. Kim J. J. Wakatsuki H. (1996). Deficient cerebellar long-term depression, impaired eyeblink conditioning, and normal motor coordination in GFAP mutant mice. *Neuron* 16 587–599. 10.1016/s0896-6273(00)80078-1 8785056

[B58] Sosunov A. A. Wu X. Tsankova N. M. Guilfoyle E. McKhann G. M. II Goldman J. E. (2014). Phenotypic heterogeneity and plasticity of isocortical and hippocampal astrocytes in the human brain. *J. Neurosci.* 34 2285–2298. 10.1523/JNEUROSCI.4037-13.2014 24501367 PMC3913872

[B59] Srinivasan R. Lu T. Y. Chai H. Xu J. Huang B. S. Golshani P. (2016). New transgenic mouse lines for selectively targeting astrocytes and studying calcium signals in astrocyte processes in situ and in vivo. *Neuron* 92 1181–1195. 10.1016/j.neuron.2016.11.030 27939582 PMC5403514

[B60] Stogsdill J. A. Harwell C. C. Goldman S. A. (2023). Astrocytes as master modulators of neural networks: Synaptic functions and disease-associated dysfunction of astrocytes. *Ann. N. Y. Acad. Sci.* 1525 41–60. 10.1111/nyas.15004 37219367

[B61] Verkhratsky A. Parpura V. Li B. Scuderi C. (2021). Astrocytes: The housekeepers and guardians of the CNS. *Adv. Neurobiol.* 26 21–53. 10.1007/978-3-030-77375-5_2 34888829 PMC9004589

[B62] Viana J. F. Machado J. L. Abreu D. S. Veiga A. Barsanti S. Tavares G. (2023). Astrocyte structural heterogeneity in the mouse hippocampus. *Glia* 71 1667–1682. 10.1002/glia.24362 36949723

[B63] Wilhelmsson U. Li L. Pekna M. Berthold C. H. Blom S. Eliasson C. (2004). Absence of glial fibrillary acidic protein and vimentin prevents hypertrophy of astrocytic processes and improves post-traumatic regeneration. *J. Neurosci.* 24 5016–5021. 10.1523/JNEUROSCI.0820-04.2004 15163694 PMC6729371

[B64] Winchenbach J. Düking T. Berghoff S. A. Stumpf S. K. Hülsmann S. Nave K. A. (2016). Inducible targeting of CNS astrocytes in Aldh1l1-CreERT2 BAC transgenic mice. *F1000Research* 5:2934. 10.12688/f1000research.10509.1 28149504 PMC5265728

[B65] Wruck W. Adjaye J. (2020). Meta-analysis of human prefrontal cortex reveals activation of GFAP and decline of synaptic transmission in the aging brain. *Acta Neuropathol. Commun.* 8:26. 10.1186/s40478-020-00907-8 32138778 PMC7059712

[B66] Xue A. Kong R. Yang Q. Eldaief M. C. Angeli P. A. DiNicola L. M. (2021). The detailed organization of the human cerebellum estimated by intrinsic functional connectivity within the individual. *J. Neurophysiol.* 125 358–384. 10.1152/jn.00561.2020 33427596 PMC7948146

[B67] Yamada K. Fukaya M. Shibata T. Kurihara H. Tanaka K. Inoue Y. (2000). Dynamic transformation of Bergmann glial fibers proceeds in correlation with dendritic outgrowth and synapse formation of cerebellar Purkinje cells. *J. Comp. Neurol.* 418 106–120. 10.1002/(SICI)1096-9861(20000228)418:1<106::AID-CNE8<3.0.CO;2-N 10701759

[B68] Yang Y. Vidensky S. Jin L. Jie C. Lorenzini I. Frankl M. (2011). Molecular comparison of GLT1+ and ALDH1L1+ astrocytes in vivo in astroglial reporter mice. *Glia* 59 200–207. 10.1002/glia.21089 21046559 PMC3199134

[B69] Yang Z. Wang K. K. (2015). Glial fibrillary acidic protein: From intermediate filament assembly and gliosis to neurobiomarker. *Trends Neurosci.* 38 364–374. 10.1016/j.tins.2015.04.003 25975510 PMC4559283

[B70] Zhang Y. Sloan S. A. Clarke L. E. Caneda C. Plaza C. A. Blumenthal P. D. (2016). Purification and characterization of progenitor and mature human astrocytes reveals transcriptional and functional differences with mouse. *Neuron* 89 37–53. 10.1016/j.neuron.2015.11.013 26687838 PMC4707064

