# Supplementary material for: Distribution and morphological features of astrocytes and Purkinje cells in the human cerebellum
Source: Front Neuroanat. 2025 Jul 4;19:1592671. doi: 10.3389/fnana.2025.1592671 (PMC12271190; doi:10.3389/fnana.2025.1592671)
Supplement: Supplementary file 1 [file Table_1.docx]

| **Supplemental Table 1. Stereological Parameters in the Human Cerebellum** | | | | | | |
| --- | --- | --- | --- | --- | --- | --- |
| **Species** | **Lobule** | **Layer** | **Probe** | **Counting Frame** | **Grid Size** | **Number of Sections** |
|  |  |  |  | **(µm)** | **(µm)** |  |
| Human | III, crus I, vermis | PCL, WM | Cavalieri |  | 50X50 | 4-7 |
| Human | III, crus I, vermis | GCL | Cavalieri |  | 100x100 | 4-7 |
| Human | III, crus I, vermis | PCL | OF_Astro | 50X50 | 189X189 | III = 5-7; crus I = 4-6; vermis = 6 |
| Human | III, crus I, vermis | GCL | OF_Astro | 50X50 | 500X500 | III = 5-7; crus I = 4-6; vermis = 6 |
| Human | III, crus I, vermis | WM | OF_Astro | 50X50 | 354X354 | III = 5-7; crus I = 4-6; vermis = 6 |
| Human | III, crus I, vermis | PCL | OF_Pcells | 70X70 | 106X106 | 4-7 |
| Astro astrocytes, GCL granule cell layer, OF optical fractionator, Pcells Purkinje cells, PCL Purkinje cell layer, | | | | | | |
| WM white matter | |  |  |  |  |  |

| **Supplemental Table 2. Stereological Parameters in Mice and Macaque Cerebellum** | | | | | | |
| --- | --- | --- | --- | --- | --- | --- |
| **Species** | **Lobule** | **Layer** | **Probe** | **Counting Frame** | **Grid Size** | **Number of Sections** |
|  |  |  |  | **(µm)** | **(µm)** |  |
| mouse 1&2 | III, crus I, vermis | PCL, GCL, WM | Cavalieri |  | 50X50 | 4-7 |
| mouse 1&2 | III | PCL | OF_Pcells | 40X40 | 46X46 | 6-7 |
| mouse 1&2 | crus I | PCL | OF_Pcells | 40X40 | 57X57 | 5-6 |
| mouse 1&2 | vermis | PCL | OF_Pcells | 40X40 | 40X40 | 4 |
| mouse 1 | III | PCL | OF_Astro | 40X40 | 136X101 | 7 |
| mouse 1 | III | GCL | OF_Astro | 50X50 | 136X101 | 7 |
| mouse 1 | III | WM | OF_Astro | 75X75 | 136X101 | 7 |
| mouse 1 | crus I | PCL | OF_Astro | 40X40 | 103X103 | 6 |
| mouse 1 | vermis | PCL | OF_Astro | 40X40 | 40X40 | 4 |
| mouse 1 | vermis | WM | OF_Astro | 50X50 | 54X54 | 4 |
| mouse 1&2 | crusI | GCL | OF_Astro | 50X50 | 139X139 | 5-6 |
| mouse 1&2 | crus I | WM | OF_Astro | 75X75 | 106X106 | 5-6 |
| mouse 1&2 | vermis | GCL | OF_Astro | 50X50 | 62X62 | 4 |
| mouse 2 | III | PCL | OF_Astro | 40X40 | 141X141 | 6 |
| mouse 2 | III | GCL | OF_Astro | 50X50 | 144X144 | 6 |
| mouse 2 | III | WM | OF_Astro | 75X75 | 150X150 | 6 |
| mouse 2 | crus I | PCL | OF_Astro | 40X40 | 141X141 | 5 |
| mouse 2 | vermis | PCL | OF_Astro | 40X40 | 52X52 | 4 |
| mouse 2 | vermis | WM | OF_Astro | 50X50 | 62X62 | 4 |
| macaque | III, crus I, vermis | PCL, WM | Cavalieri |  | 50X50 | 3-5 |
| macaque | III, crus I, vermis | GCL | Cavalieri |  | 100x100 | 3-5 |
| macaque | III, crus I, vermis | PCL | OF_Astro | 70X70 | 181X181 | III = 4-5; crus I = 3; vermis= 3-4 |
| macaque | III, crus I, vermis | GCL | OF_Astro | 80X80 | 253X253 | III = 4-5; crus I = 3; vermis= 3-4 |
| macaque | III, crus I, vermis | WM | OF_Astro | 80X80 | 179X179 | III = 4-5; crus I = 3; vermis= 3-4 |
| macaque | III, crus I, vermis | PCL | OF_Pcells | 70X70 | 99X99 | 3-5 |
| Astro astrocytes, GCL granule cell layer, OF optical fractionator, Pcells Purkinje cells, PCL Purkinje cell layer, WM white matter | | | | | | |
